# Supplementary material for: Meta-analysis of northeast Atlantic marine taxa shows contrasting phylogeographic patterns following post-LGM expansions
Source: PeerJ. 2018 Sep 28;6:e5684. doi: 10.7717/peerj.5684 (PMC6166638; doi:10.7717/peerj.5684)
Supplement: Table S1 [file peerj-06-5684-s001.docx]

**Table S1a:** Summary of the mutation rates used for timing population expansions.

| **Species** | **mtDNA**  **gene** | **Mutation rate per Myr (%)** | **Mutation rate reference** | **Calibration date** | **Calibration event** |
| --- | --- | --- | --- | --- | --- |
| *Carcinus maenas* | COI | 6.58-8.85 | Crandall *et al.* 2012 (mantis shrimp) | 19.60 Kyr & 14.58 Kyr | Radiometric data for sea-level rise onto the Sunda Shelf post-LGM |
| *Maja brachydactyla* | COI | ʺ ʺ | ʺ ʺ | ʺ ʺ | ʺ ʺ |
| *Palinurus elephas* | COI | ʺ ʺ | ʺ ʺ | ʺ ʺ | ʺ ʺ |
| *Conger conger* | CR | 10.0-13.2 | Genner *et al.* 2007 (cichlids) | 25 Kyr & 23 Kyr | Isolation of Lake Kivu (*P* distance = 0.0033) & formation of Lake Apoyo (*P* distance = 0.0023) |
| *Dicentrarchus labrax* | CR | ʺ ʺ | ʺ ʺ | ʺ ʺ | ʺ ʺ |
| *Labrus bergylta* | CR | ʺ ʺ | ʺ ʺ | ʺ ʺ | ʺ ʺ |
| *Symphodus melops* | CR | ʺ ʺ | ʺ ʺ | ʺ ʺ | ʺ ʺ |
| *Pomatoschistus microps* | Cyt *b* | 4-6 | Larmuseau *et al.* 2009 (*Pomatoschistus* gobies) | 15-14 Myr | Closure of Atlantic-Mediterranean part of the Tethys Sea |
| *Pomatoschistus minutus* | Cyt *b* | ʺ ʺ | ʺ ʺ | ʺ ʺ | ʺ ʺ |
| *Solea solea* | Cyt *b* | ʺ ʺ | ʺ ʺ | ʺ ʺ | ʺ ʺ |
| *Cerastoderma edule* | COI | 11 | Laakkonen *et al.* 2015 (bivalve) | 3.5 Myr | Trans-Arctic divergence |
| *Modiolus modiolus* lineage 1 | COI | ʺ ʺ | ʺ ʺ | ʺ ʺ | ʺ ʺ |
| *Nassarius reticulatus* | COI | 10 | González-Wevar *et al.* 2013 (nacellids) | N/A | Authors accounted for time-dependency of molecular rates |
| *Owenia fusiformis* lineage 1 | COI | 2.2 | Jolly *et al.* 2006 (polychaetes) | 28.5 Myr | Authors based rate on Chevaldonne *et al.* (2002) study |
| *Owenia fusiformis* lineage 2 | COI | ʺ ʺ | ʺ ʺ | ʺ ʺ | ʺ ʺ |
| *Owenia fusiformis* lineage 3 | COI | ʺ ʺ | ʺ ʺ | ʺ ʺ | ʺ ʺ |
| *Pectinaria koreni* lineage 1 | COI | ʺ ʺ | ʺ ʺ | ʺ ʺ | ʺ ʺ |
| *Pectinaria koreni* lineage 2 | COI | ʺ ʺ | ʺ ʺ | ʺ ʺ | ʺ ʺ |
| *Pelvetia canaliculata* | IGS | 1.0-1.7 | Hoarau *et al.* 2007 (Fucaceae) | 10-16 Myr | Divergence between *Ascophyllum nodosum* and *Fucus vesiculosus*. |

**Table S1b:** Summary of the parameters used for timing population expansions. Tau was calculated using DnaSP software.

| **Species** | **Sequence length (bp)** | **Tau (τ)** | **Mutation rate per Myr (%)** | **Expansion time estimate (min-max)** |
| --- | --- | --- | --- | --- |
| *Carcinus maenas* | 502 | 1.605 | 6.58-8.85 | 21.17 (18.06-24.29) |
| *Maja brachydactyla* | 548 | 2.185 | ʺ ʺ | 26.41 (22.53-30.30) |
| *Palinurus elephas* | 499 | 0.750 | ʺ ʺ | 9.96 (8.49-11.42) |
| *Conger conger* | 607 | 2.199 | 10.0-13.2 | 15.91 (13.72-18.11) |
| *Dicentrarchus labrax* | 380 | 1.740 | ʺ ʺ | 20.12 (17.34-22.89) |
| *Labrus bergylta* | 332 | 3.900 | ʺ ʺ | 51.61 (44.5-58.73) |
| *Symphodus melops* | 364 | 1.538 | ʺ ʺ | 18.56 (16.00-21.13) |
| *Pomatoschistus microps* | 289 | 0.963 | 4-6 * | 11.57 (9.26-13.88) |
| *Pomatoschistus minutus* | 850 | 3.607 | ʺ ʺ | 14.73 (11.79-17.68) |
| *Solea solea* | 590 | 1.520 | ʺ ʺ | 8.95 (7.16-10.73) |
| *Cerastoderma edule* | 582 | 1.781 | 11 (10-12) | 14.03 (12.75-15.30) |
| *Modiolus modiolus* lineage 1 | 598 | 2.089 | ʺ ʺ | 16.02 (14.56-17.47) |
| *Nassarius reticulatus* | 395 | 0.794 | 10 (9-11) | 10.16 (9.14-11.17) |
| *Owenia fusiformis* lineage 1 | 506 | 1.977 | 2.2 (1.87-2.53) * | 30.29 (25.70-34.88) |
| *Owenia fusiformis* lineage 2 | 506 | 2.607 | ʺ ʺ | 39.95 (33.90-46.00) |
| *Owenia fusiformis* lineage 3 | 506 | 1.413 | ʺ ʺ | 21.65 (18.37-24.93) |
| *Pectinaria koreni* lineage 1 | 586 | 1.710 | ʺ ʺ | 22.62 (19.20-26.05) |
| *Pectinaria koreni* lineage 2 | 586 | 1.240 | ʺ ʺ | 16.41 (13.92-18.89) |
| *Pelvetia canaliculata* | 673 | 1.214 | 1.0-1.7 * | 25.59 (18.95-32.22) |

*A three-fold correction was applied (see main text for details)

**References**

Chevaldonne P, Jollivet D, Desbruyères D, Lutz R, Vrijenhoek RC (2002) Sister-species of eastern Pacific hydrothermal-vent worms (Ampharetidae, Alvinelidae, Vestimentifera) provide new mitochondrial clock calibration. *Cahiers de Biologie Marine*, **43**, 367–370.

Crandall ED, Sbrocco EJ, DeBoer TS, Barber PH, Carpenter KE (2012) Expansion dating: calibrating molecular clocks in marine species from expansions onto the Sunda Shelf following the Last Glacial Maximum. *Molecular Biology and Evolution*, **29**, 707–719.

Genner MJ, Seehausen O, Lunt DH *et al.* (2007) Age of cichlids: new dates for ancient lake fish radiations. *Molecular Biology and Evolution*, **24**, 1269–1282.

González-Wevar CA, Saucède T, Morley SA, Chown SL, Poulin E (2013) Extinction and recolonization of maritime Antarctica in the limpet *Nacella concinna* (Strebel, 1908) during the last glacial cycle: toward a model of Quaternary biogeography in shallow Antarctic invertebrates. *Molecular Ecology*, **22**, 5221–5236.

Hoarau G, Coyer JA, Veldsink JH, Stam WT, Olsen JL (2007) Glacial refugia and recolonization pathways in the brown seaweed *Fucus serratus*. *Molecular Ecology*, **16**, 3606–3616.

Jolly MT, Viard F, Gentil F, Thiébaut E, Jollivet D (2006) Comparative phylogeography of two coastal polychaete tubeworms in the Northeast Atlantic supports shared history and vicariant events. *Molecular Ecology*, **15**, 1841–1855.

Laakkonen HM, Strelkov P, Väinölä R (2015) Molecular lineage diversity and inter-oceanic biogeographical history in *Hiatella* (Mollusca, Bivalvia). *Zoologica Scripta*, **44**, 383–402.

Larmuseau MHD, Van Houdt JKJ, Guelinckx J, Hellemans B, Volckaert FAM (2009) Distributional and demographic consequences of Pleistocene climate fluctuations for a marine demersal fish in the north-eastern Atlantic. *Journal of Biogeography*, **36**, 1138–1151.
