# Supplementary figures and images for: Meta-analysis of northeast Atlantic marine taxa shows contrasting phylogeographic patterns following post-LGM expansions
Source: PeerJ. 2018 Sep 28;6:e5684. doi: 10.7717/peerj.5684 (PMC6166638; doi:10.7717/peerj.5684)

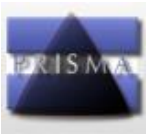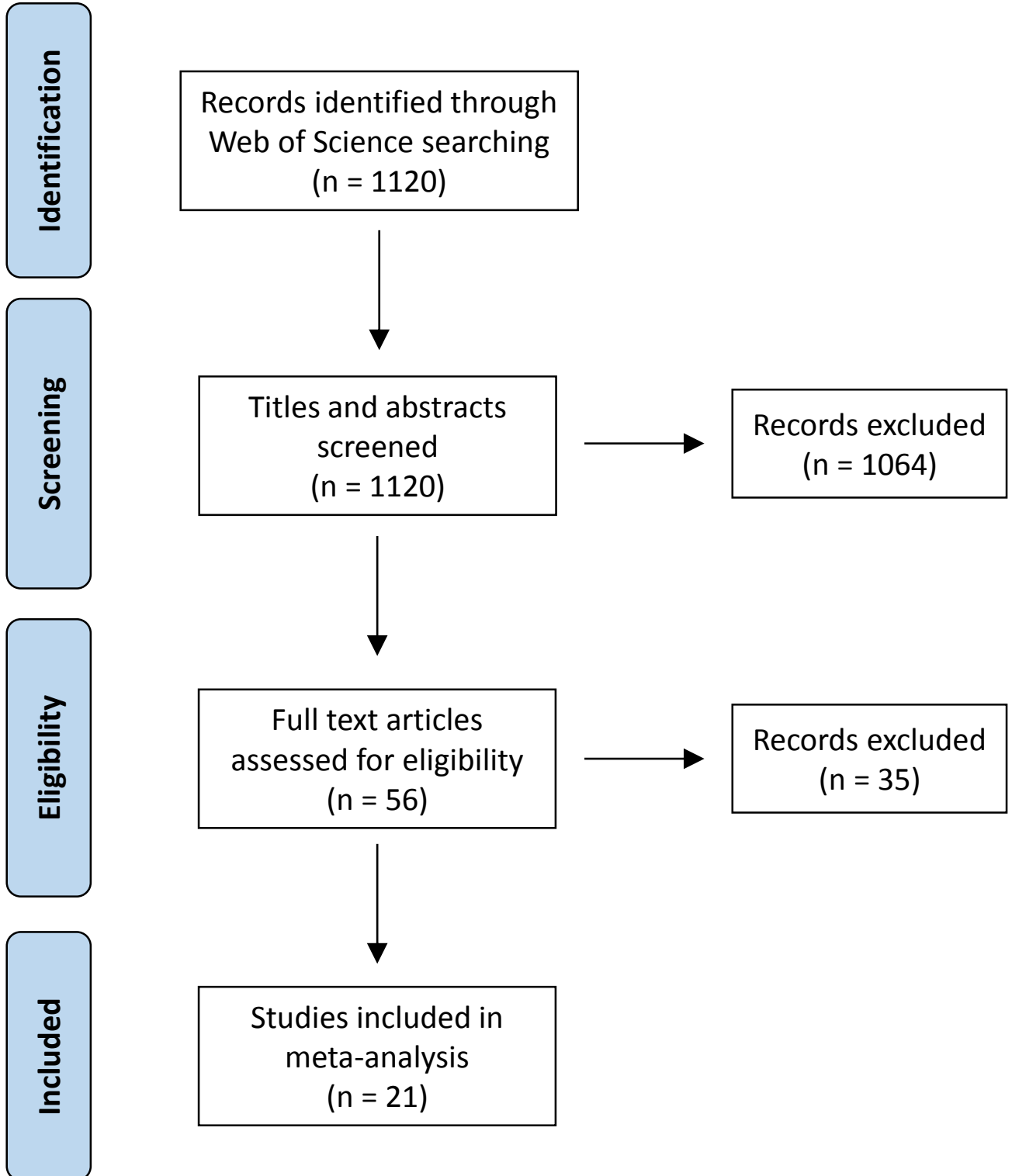

Supplement: Figure S1 [file peerj-06-5684-s003.pdf]
