# Supplementary material for: Meta-analysis of northeast Atlantic marine taxa shows contrasting phylogeographic patterns following post-LGM expansions
Source: PeerJ. 2018 Sep 28;6:e5684. doi: 10.7717/peerj.5684 (PMC6166638; doi:10.7717/peerj.5684)
Supplement: Figure S2 [file peerj-06-5684-s004.pdf]

Haplotype networks for all 21 species used in the meta-analysis
Figure S2, Supporting Information

Tom Jenkins

March 12, 2018

List of Figures

1 *Carcinus maenas* 1
2 *Celleporella hyalina* 2
3 *Cerastoderma edule* 3
4 *Conger conger* 4
5 *Dicentrarchus labrax* 5
6 *Labrus bergylta* 6
7 *Macoma balthica* 7
8 *Maja brachydactyla* 8
9 *Modiolus modiolus* 9
10 *Nassarius nitidus* 10
11 *Nassarius reticulatus* 11
12 *Neomysis integer* 12
13 *Owenia fusiformis* 13
14 *Pomatoschistus microps* 14
15 *Pomatoschistus minutus* 15
16 *Palinurus elephas* 16
17 *Pectinaria koreni* 17
18 *Pelvetia canaliculata* 18
19 *Raja clavata* 19
20 *Solea solea* 20
21 *Symphodus melops* 21

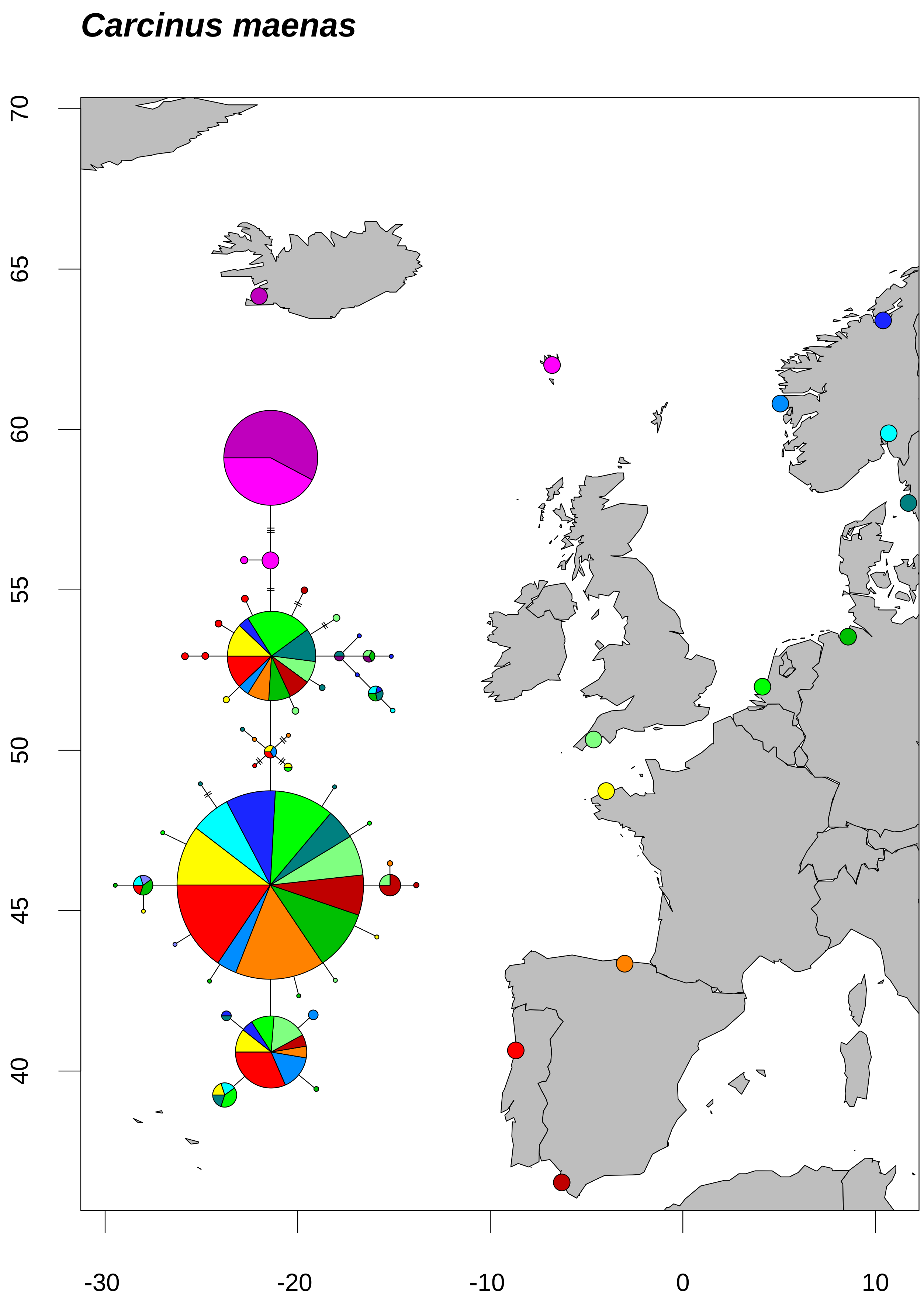

Figure 1

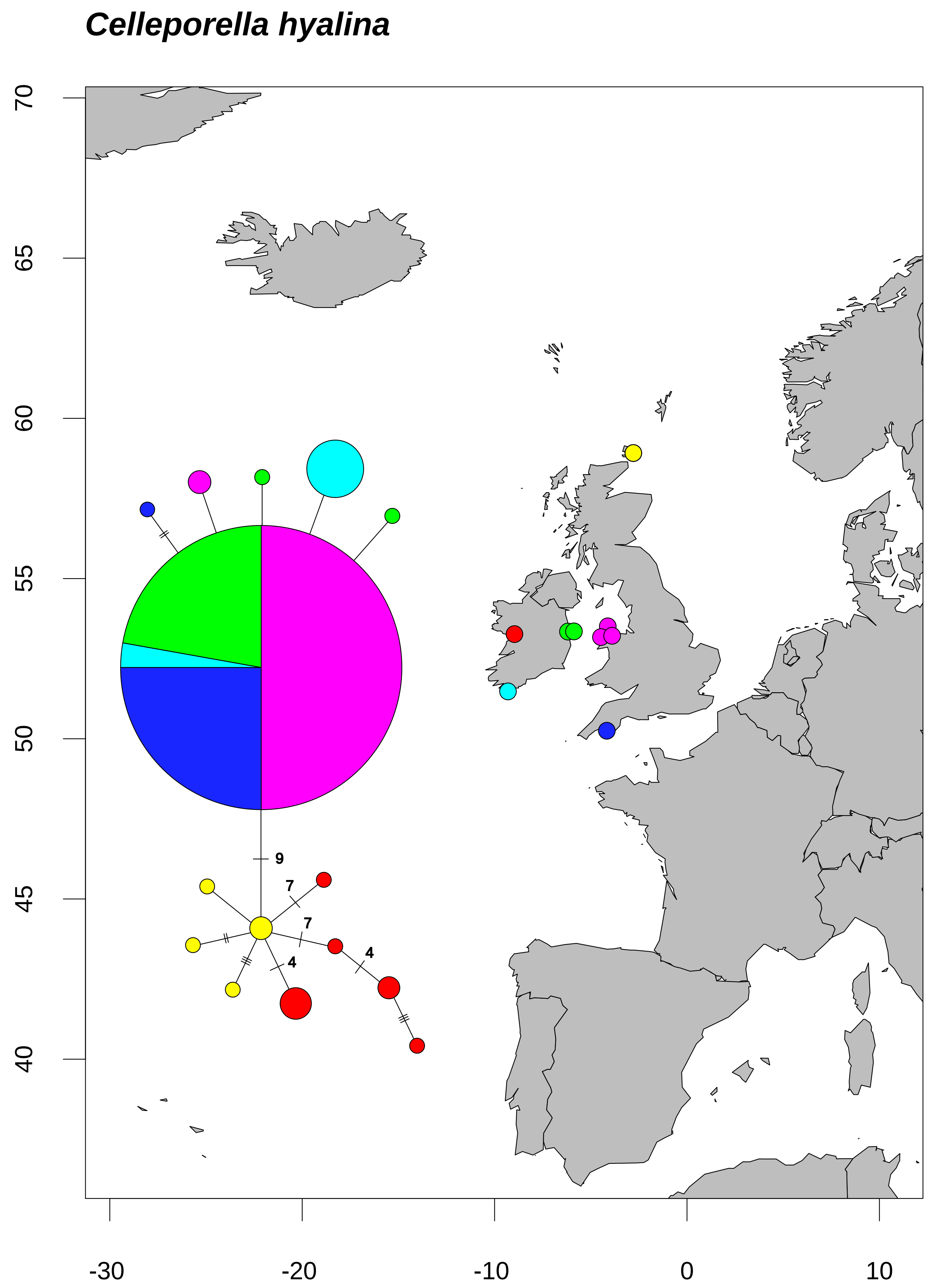

Figure 2

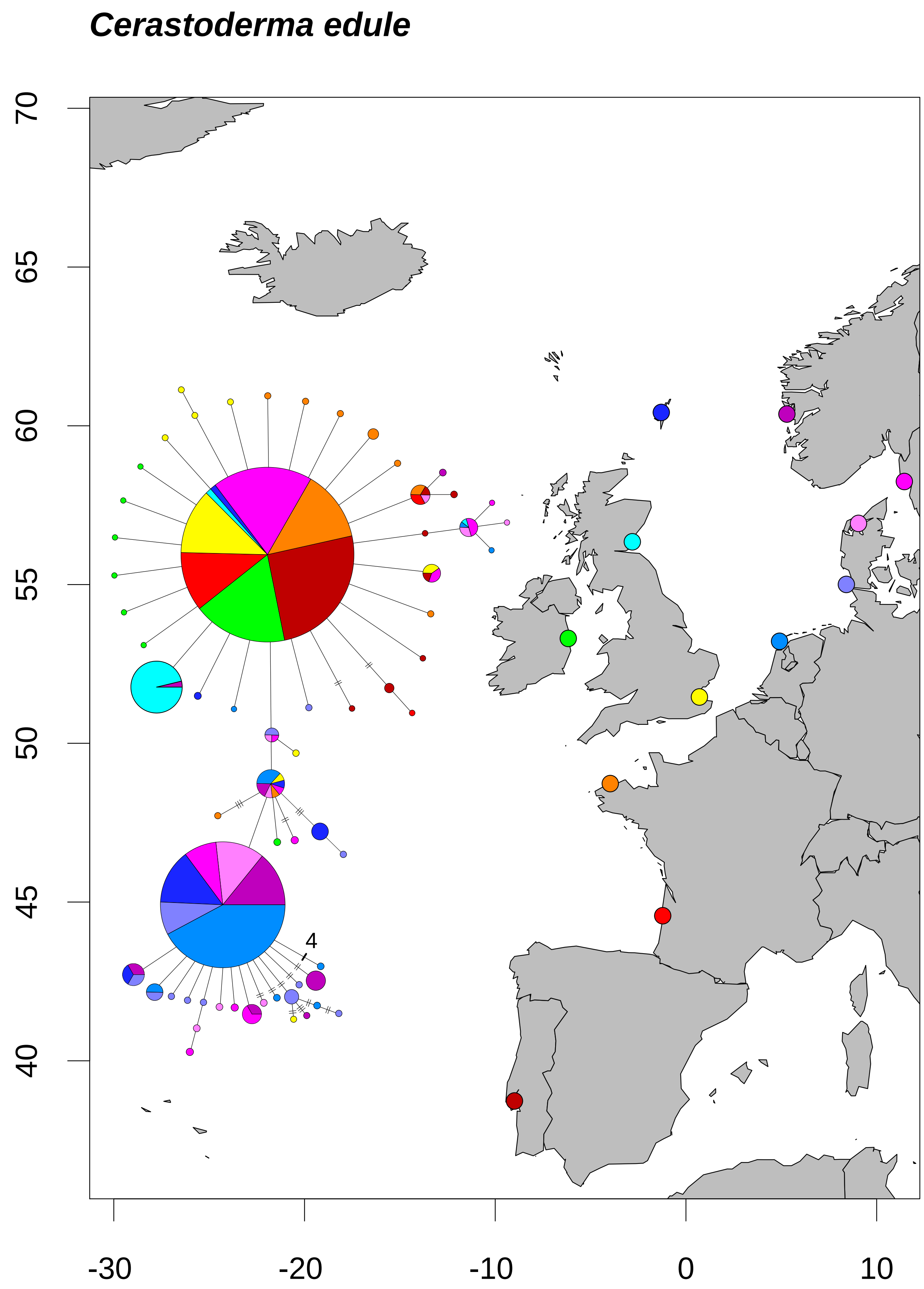

Figure 3

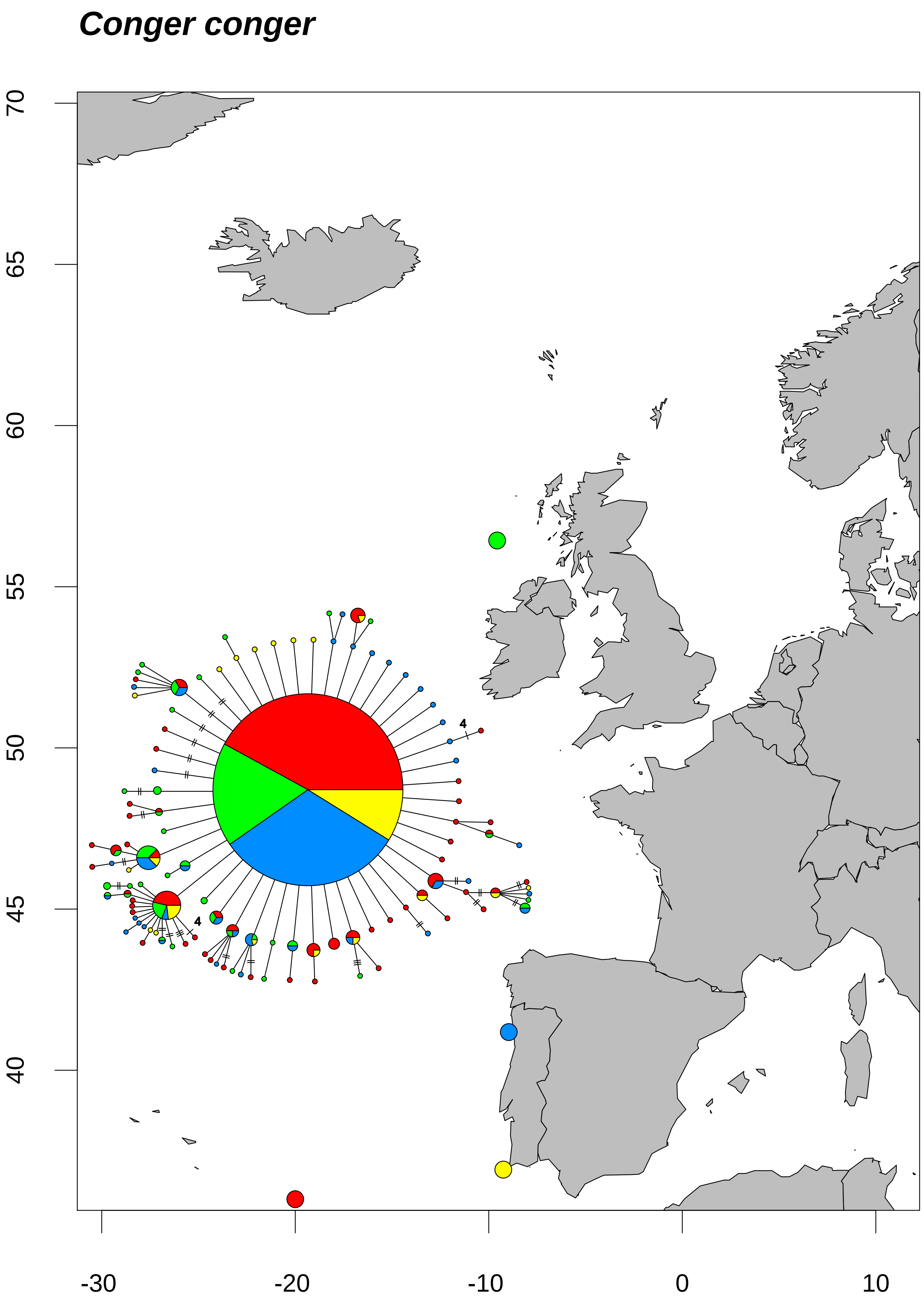

Figure 4

Dicentrarchus labrax

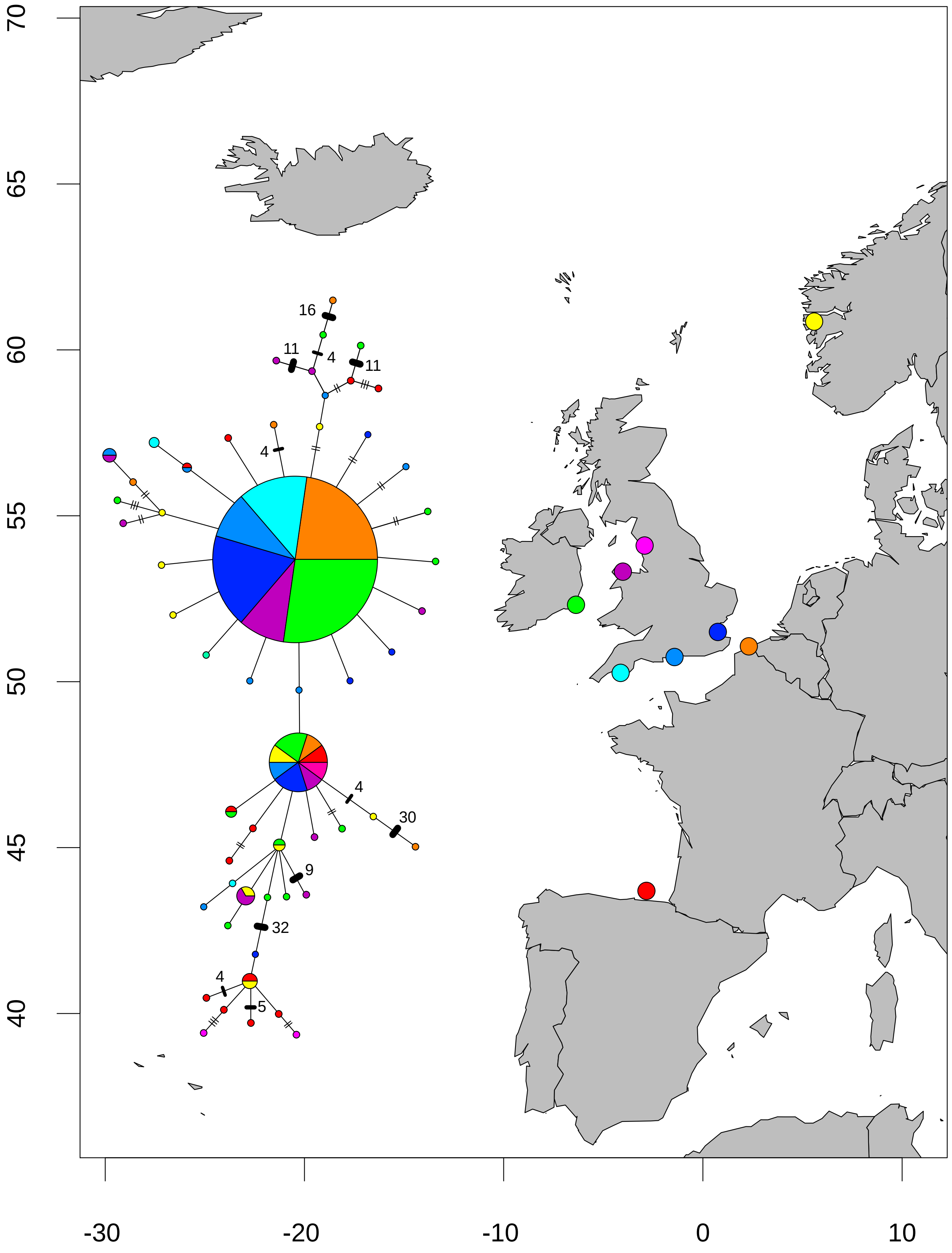

Figure 5

Labrus bergylta

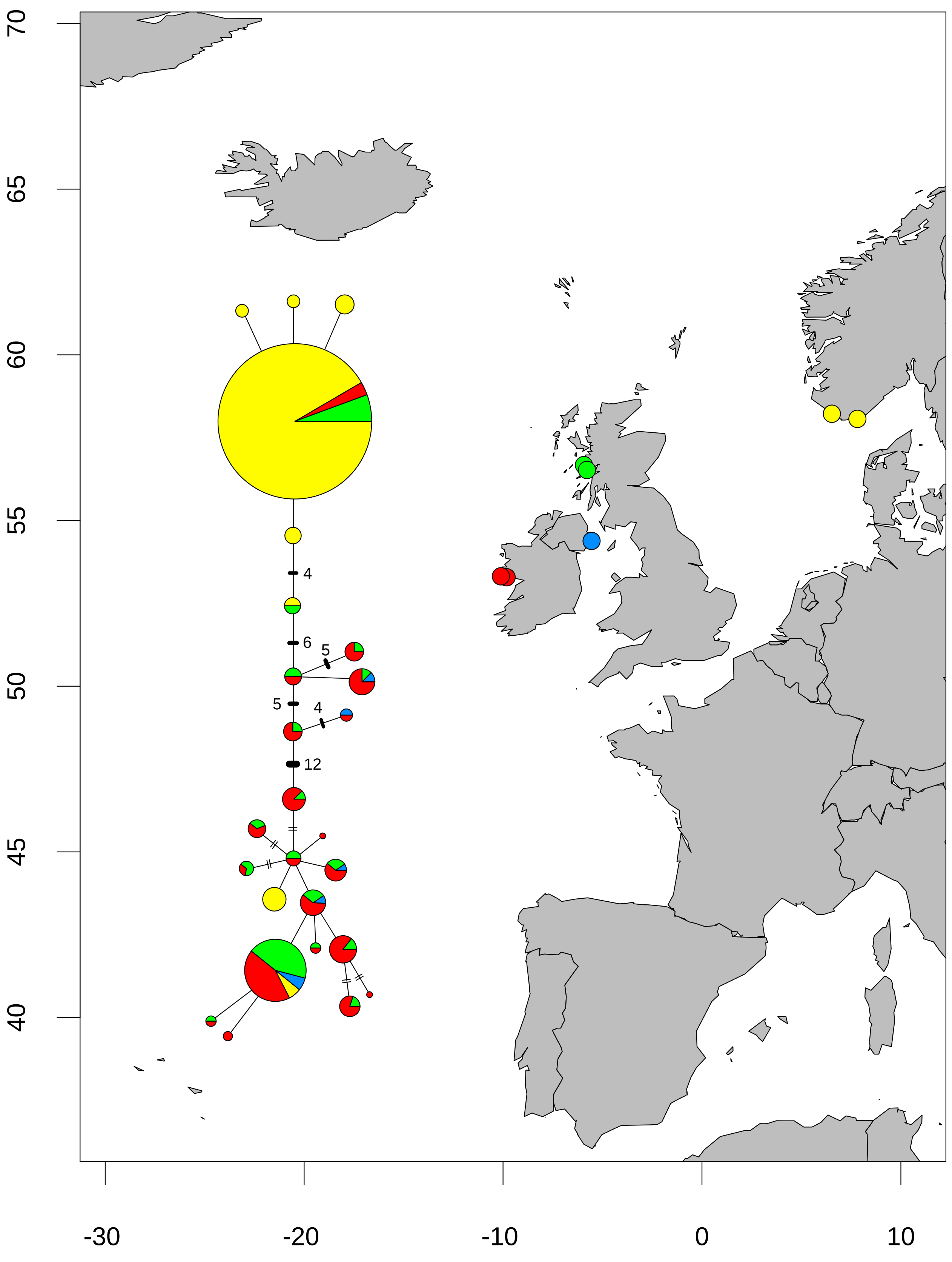

Figure 6: Only haplotypes represented by more than one sequence are shown.

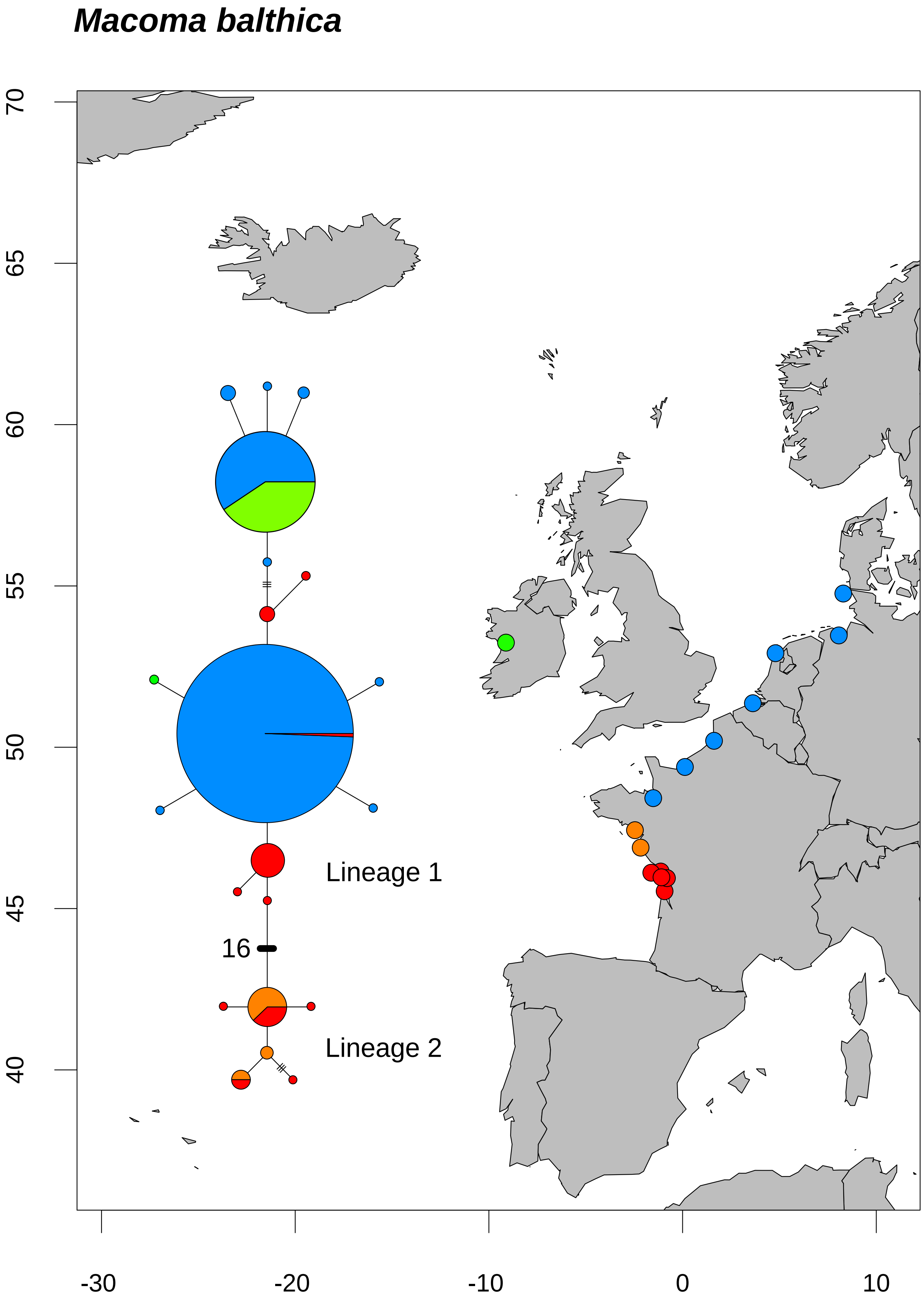
Macoma balthica
70
65
60
55
50
45
40
-30
-20
-10
0
10
Lineage 1
16
Lineage 2

Figure 7

Maja brachydactyla

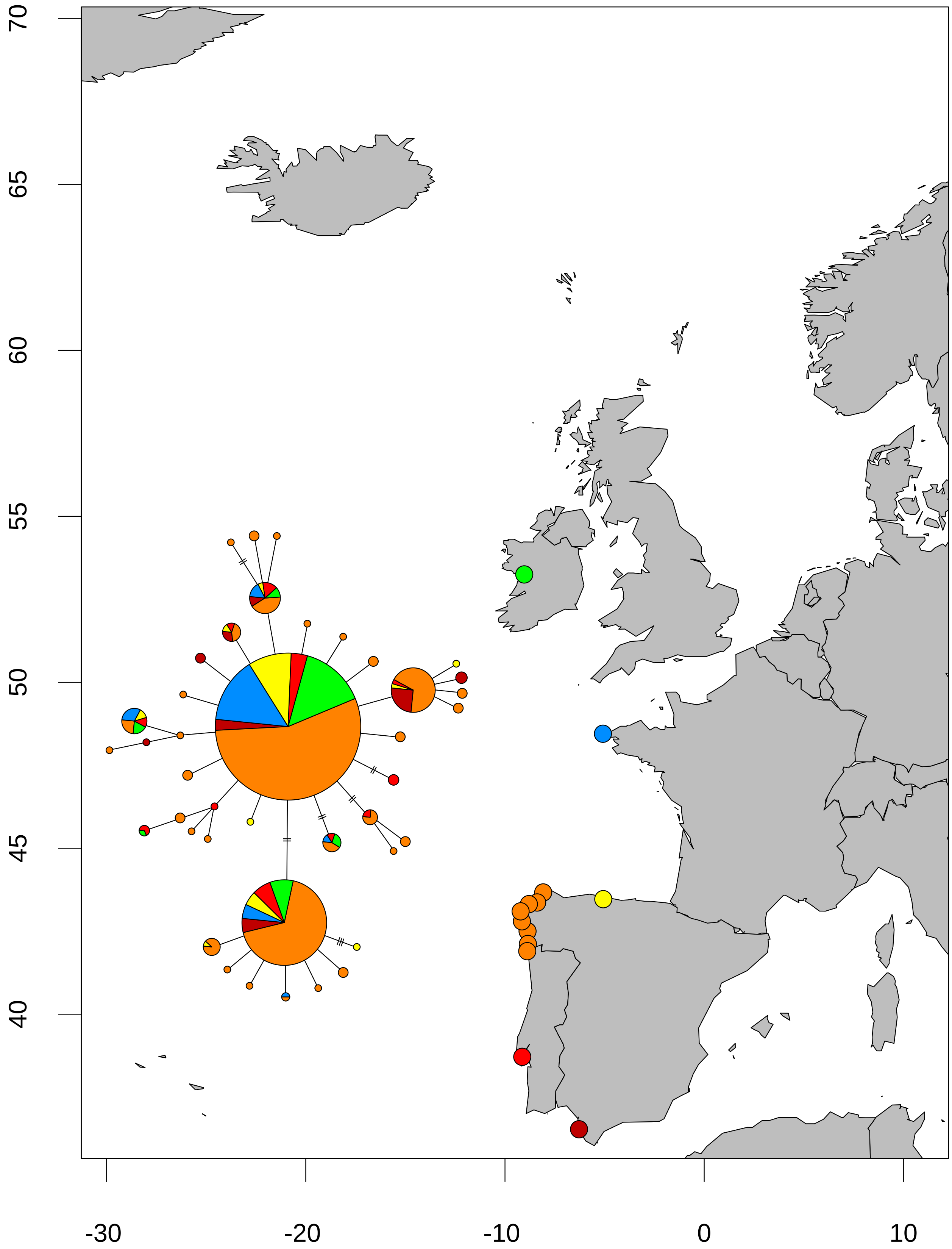

Figure 8

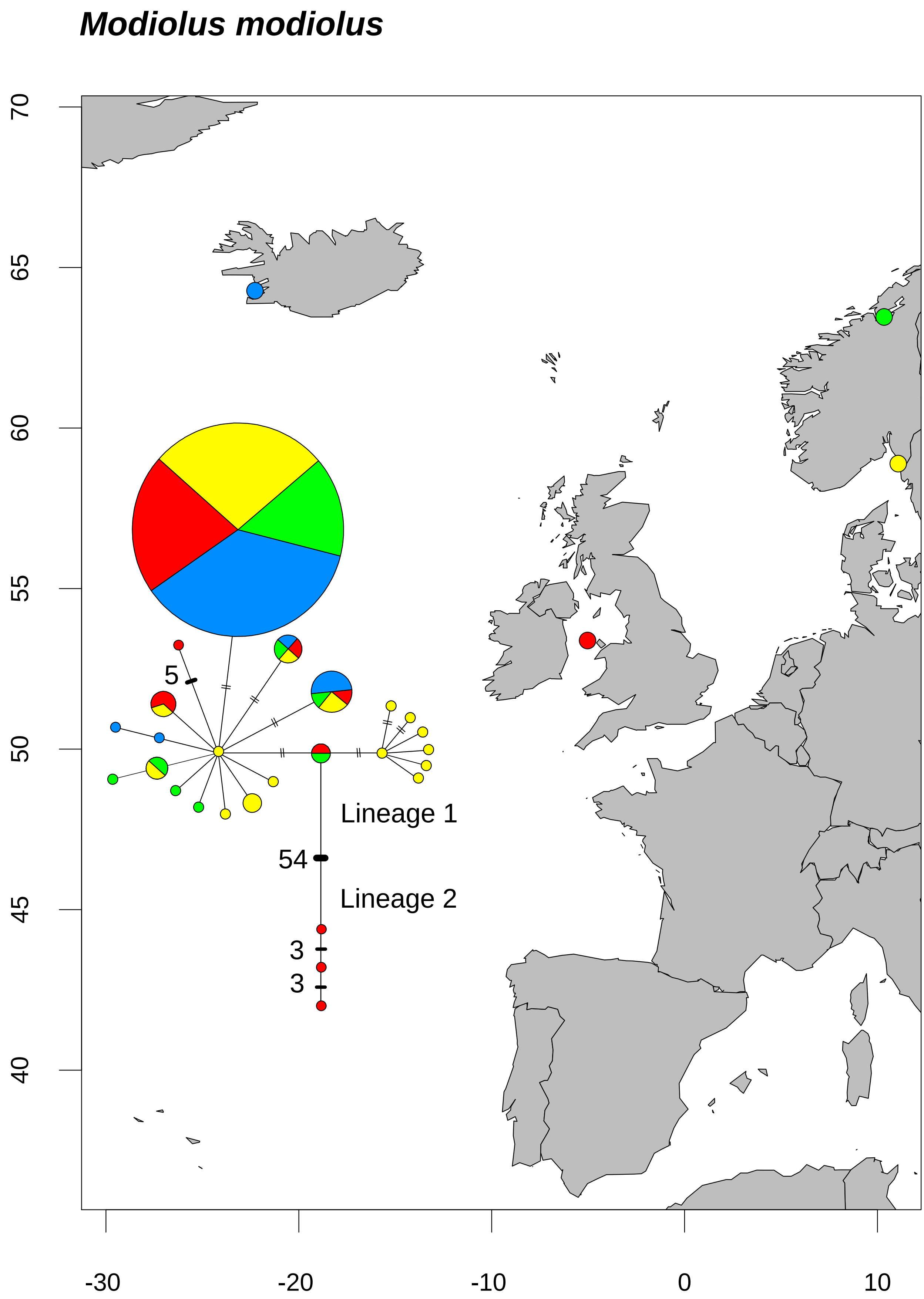
Modiolus modiolus
70
65
60
55
50
45
40
-30
-20
-10
0
10
5
Lineage 1
54
Lineage 2
3
3

Figure 9

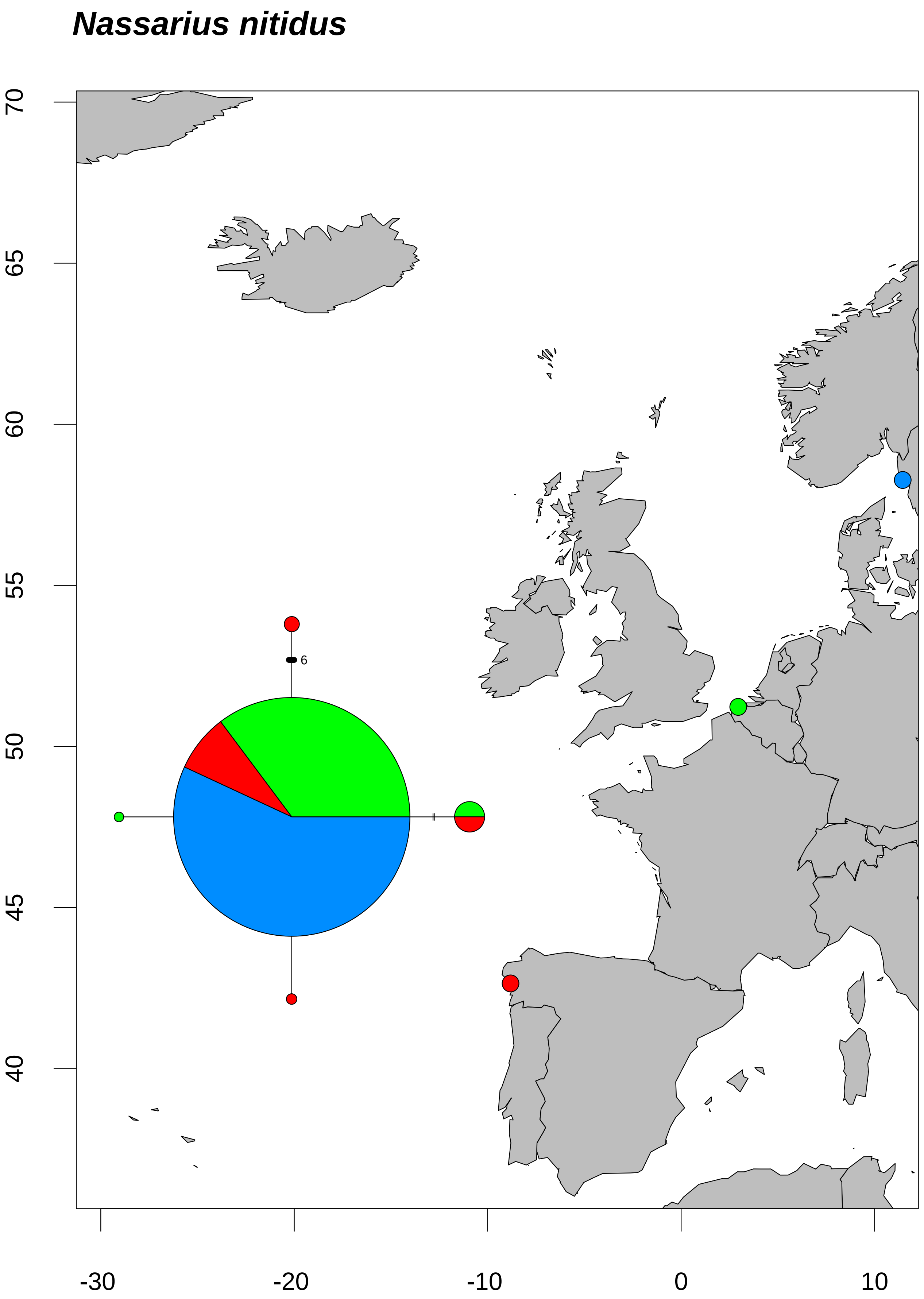

Figure 10

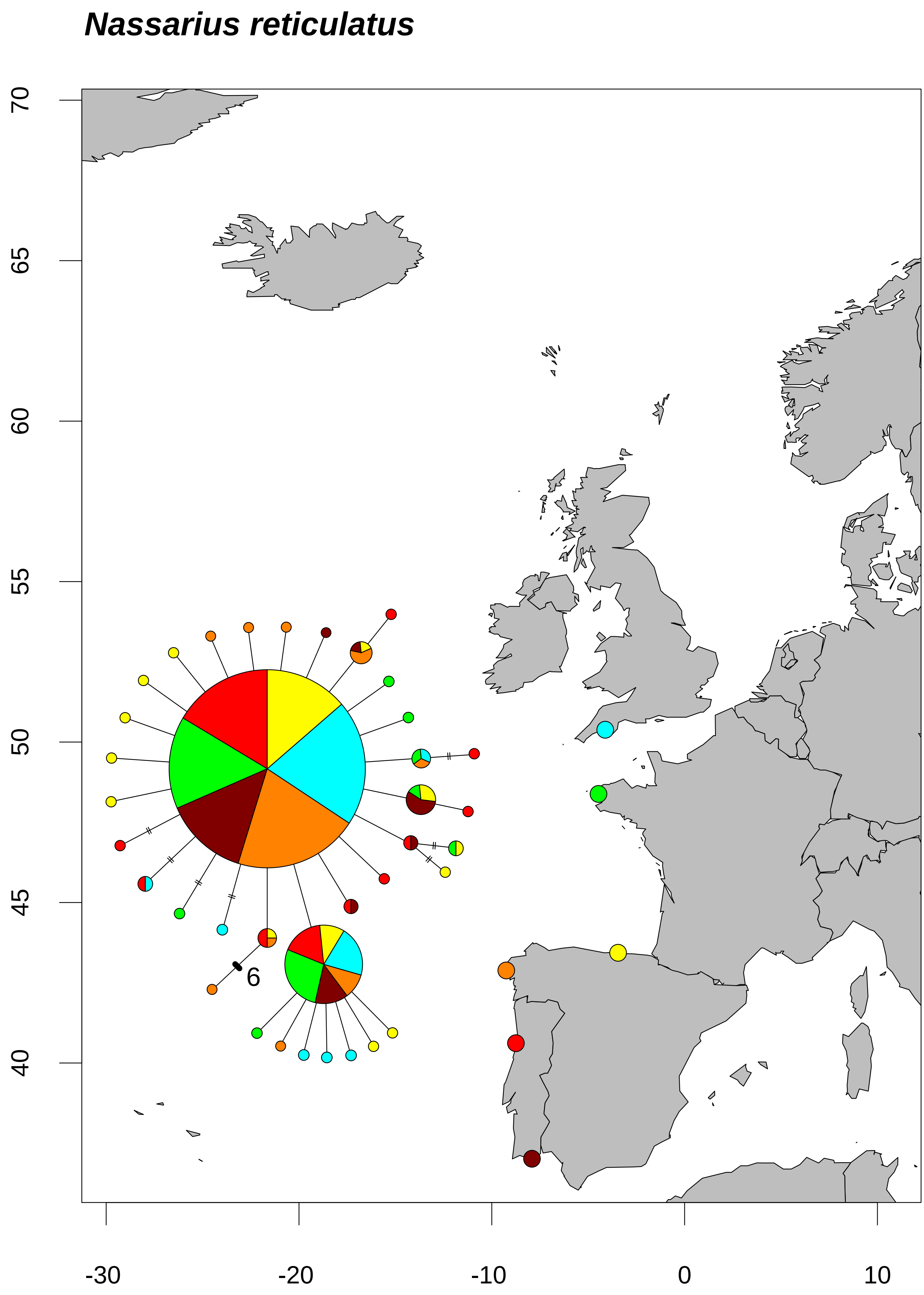

Figure 11

Neomysis integer

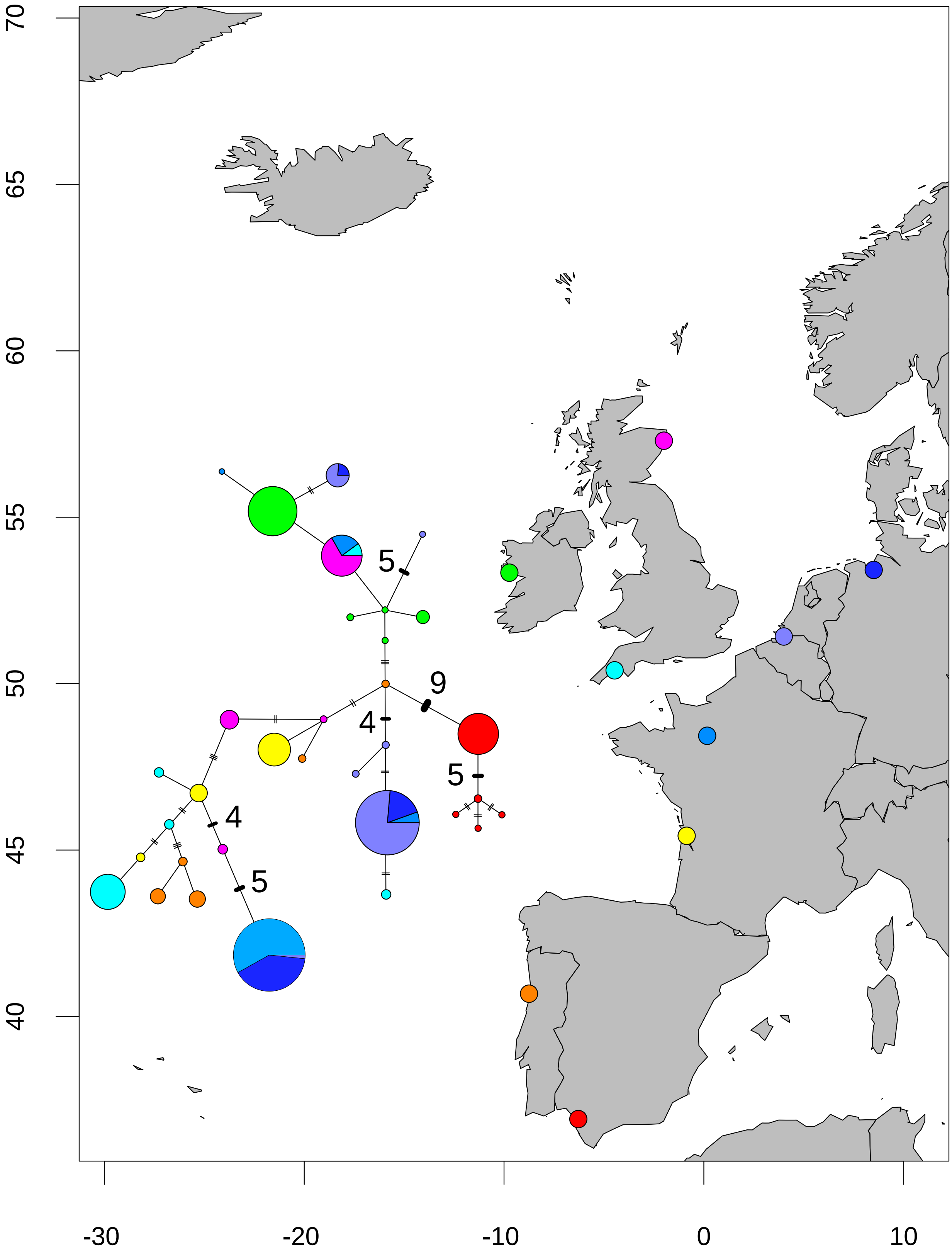

Figure 12

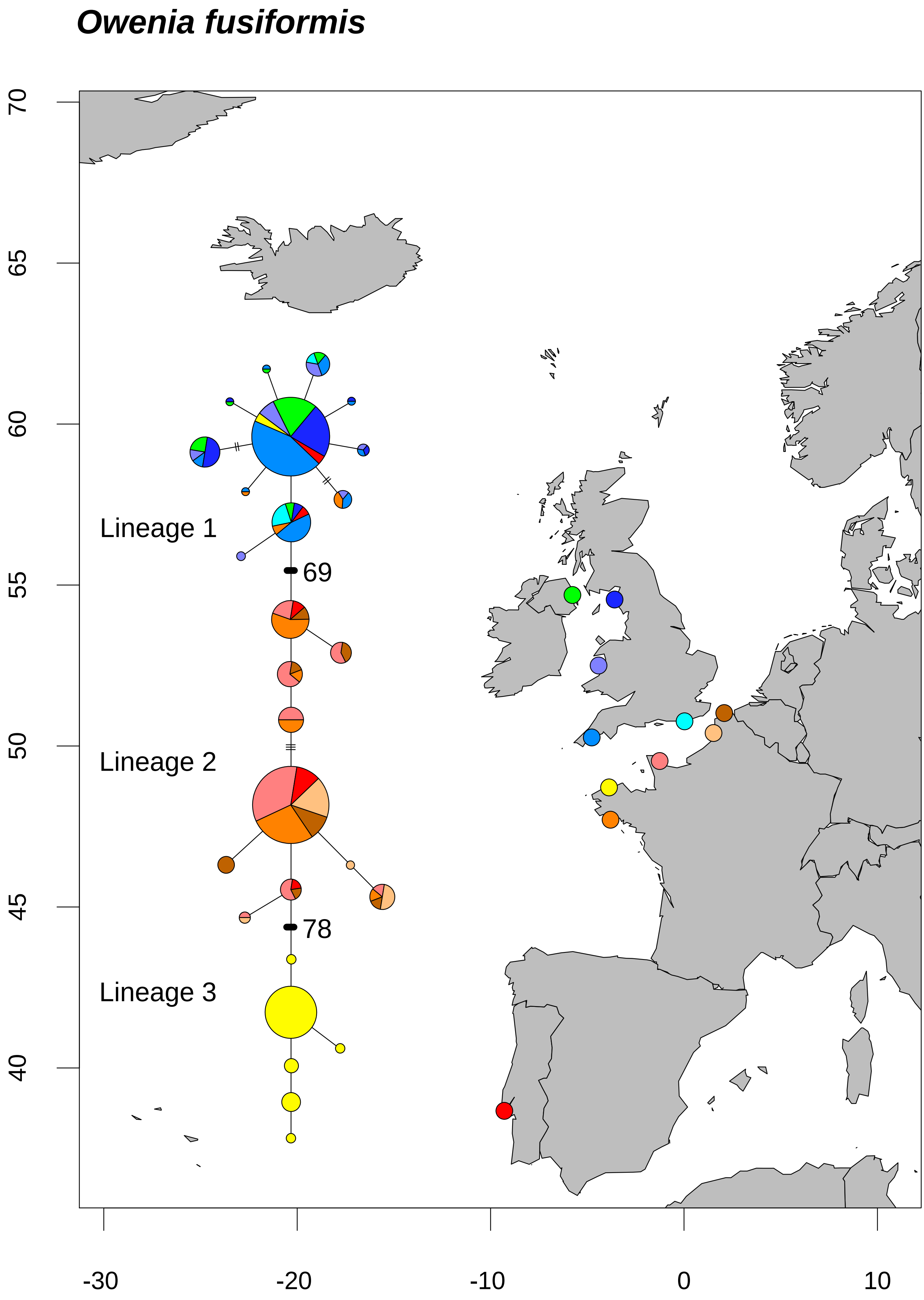

Figure 13: Only haplotypes represented by more than one sequence are shown.

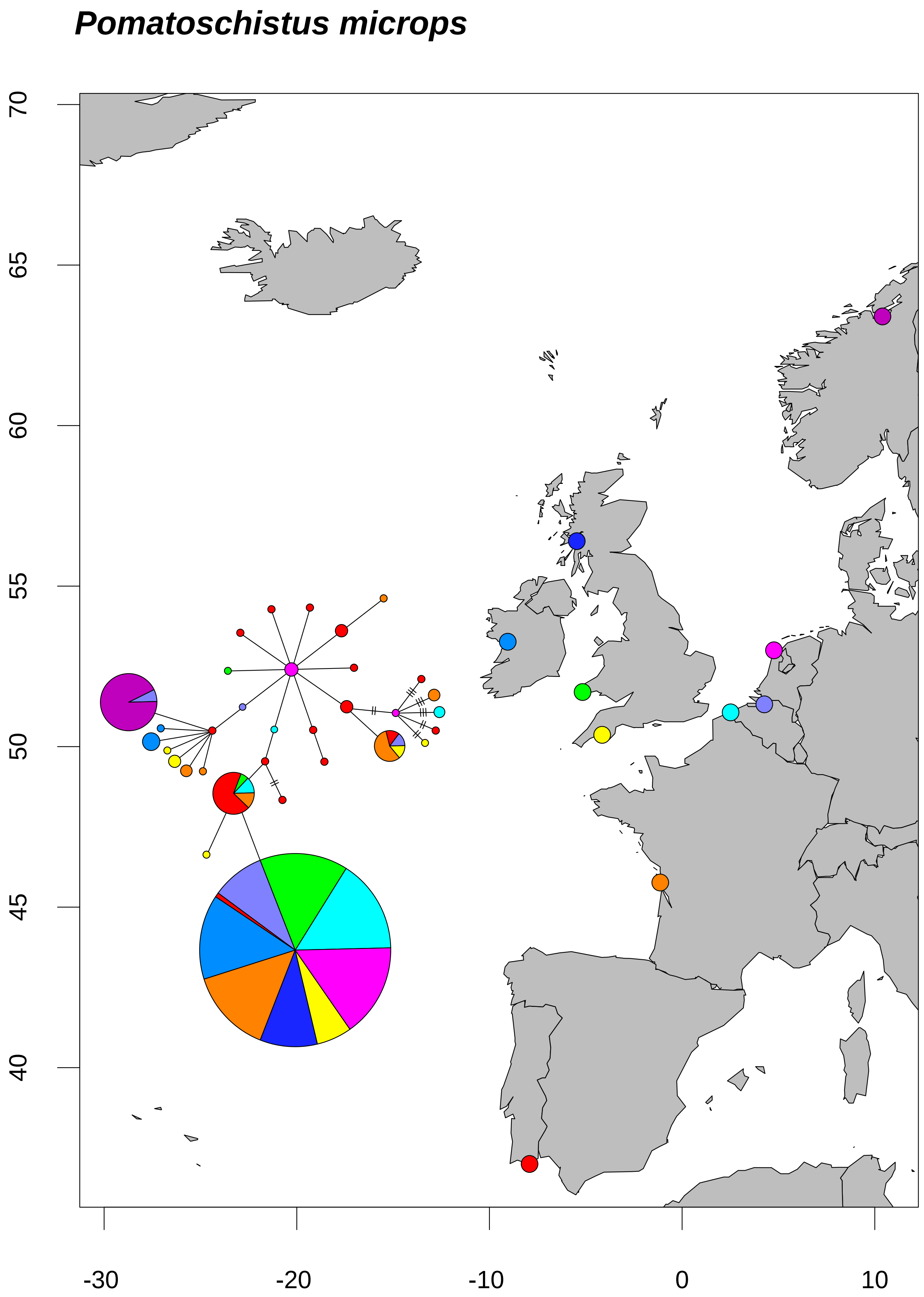

Figure 14

Pomatoschistus minutus

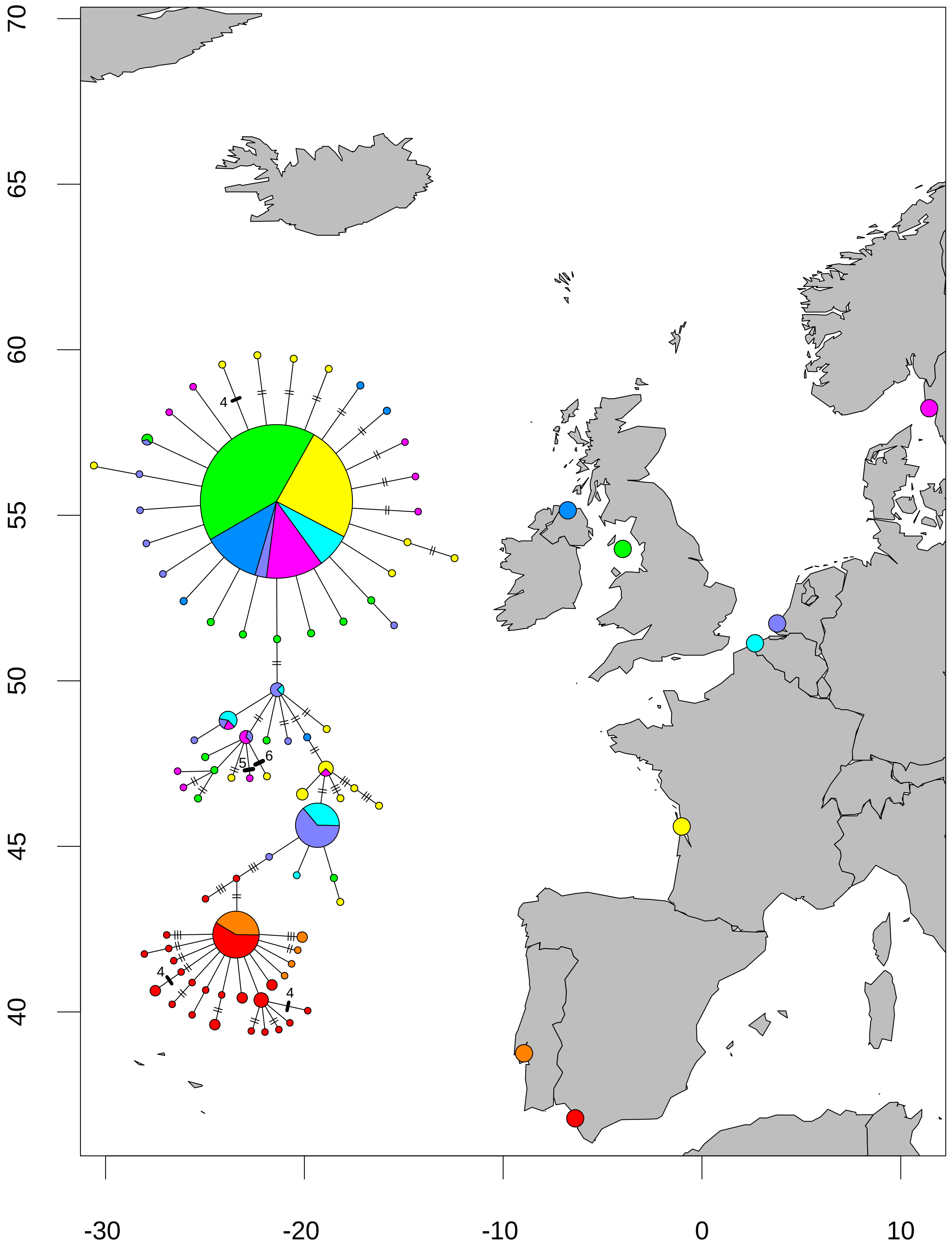

Figure 15

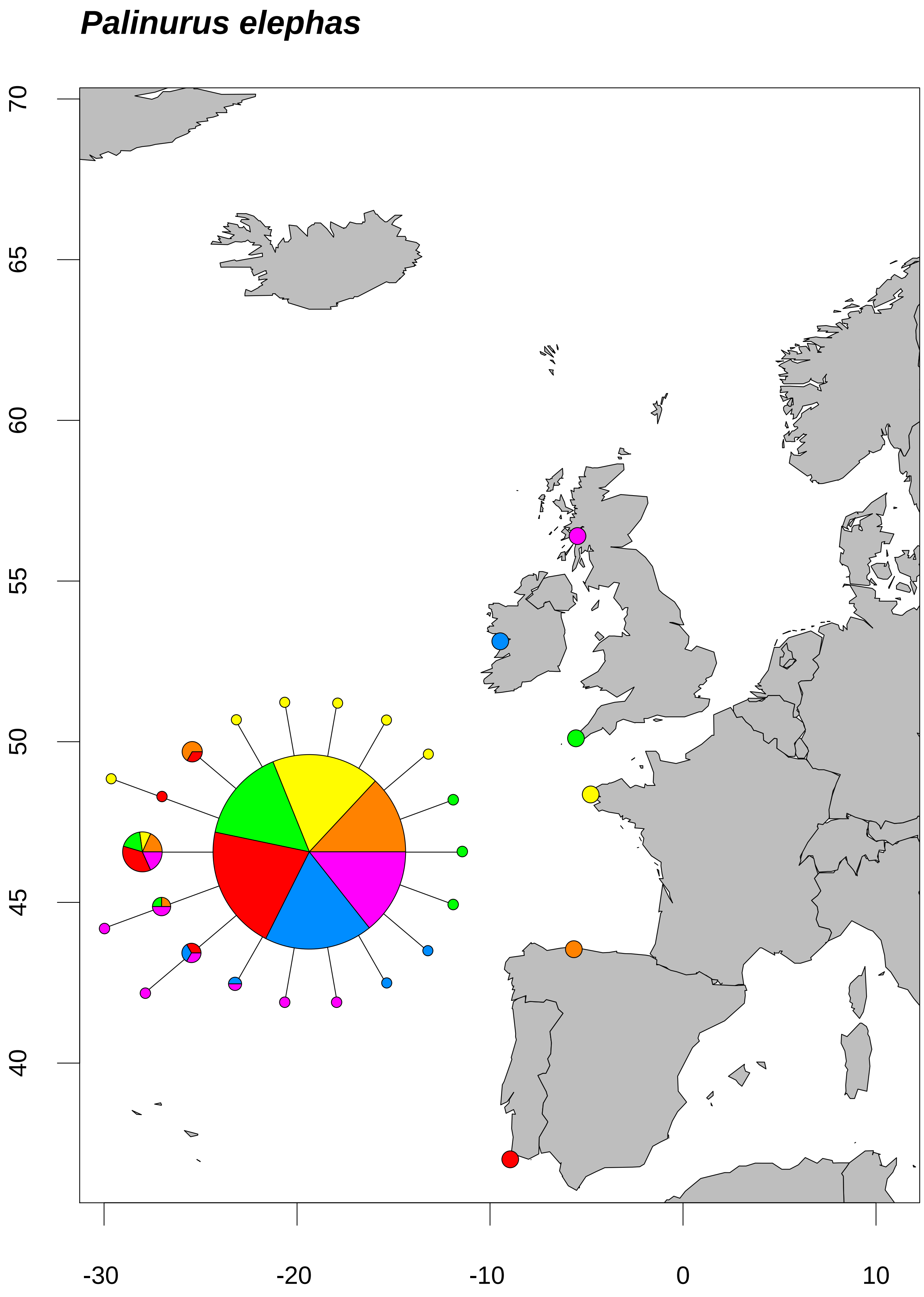

Figure 16

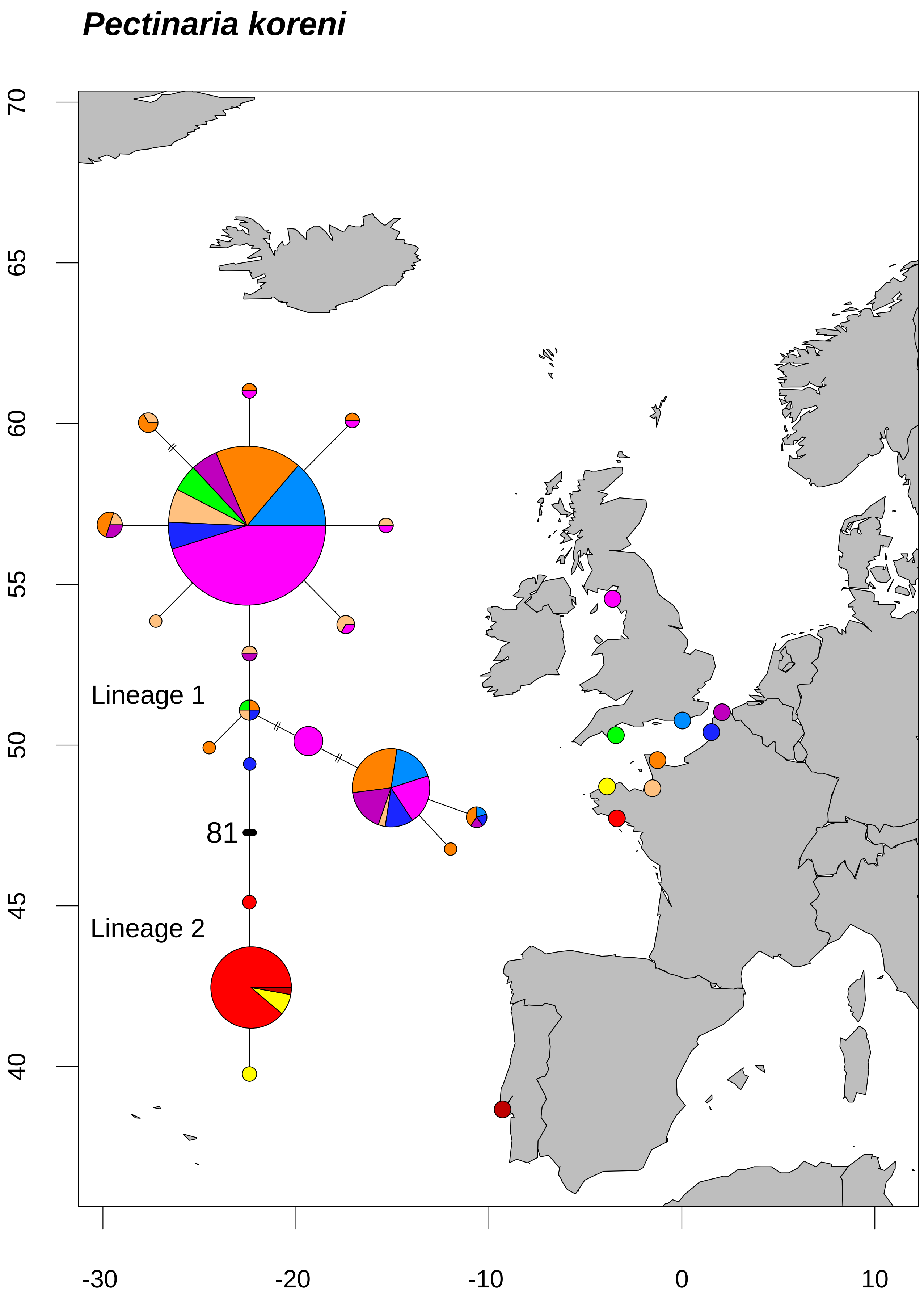

Figure 17: Only haplotypes represented by more than one sequence are shown.

Pelvetia canaliculata

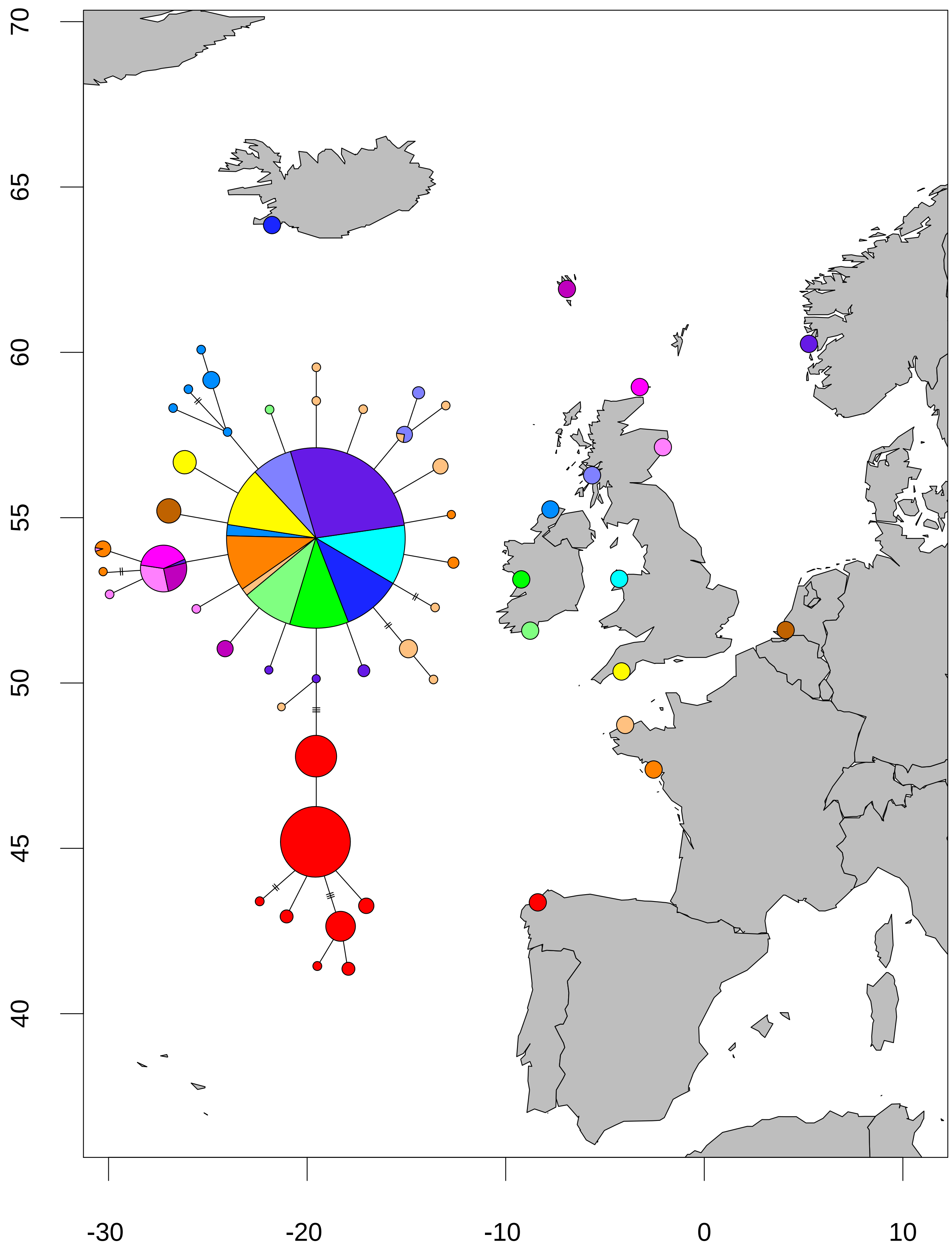

Figure 18

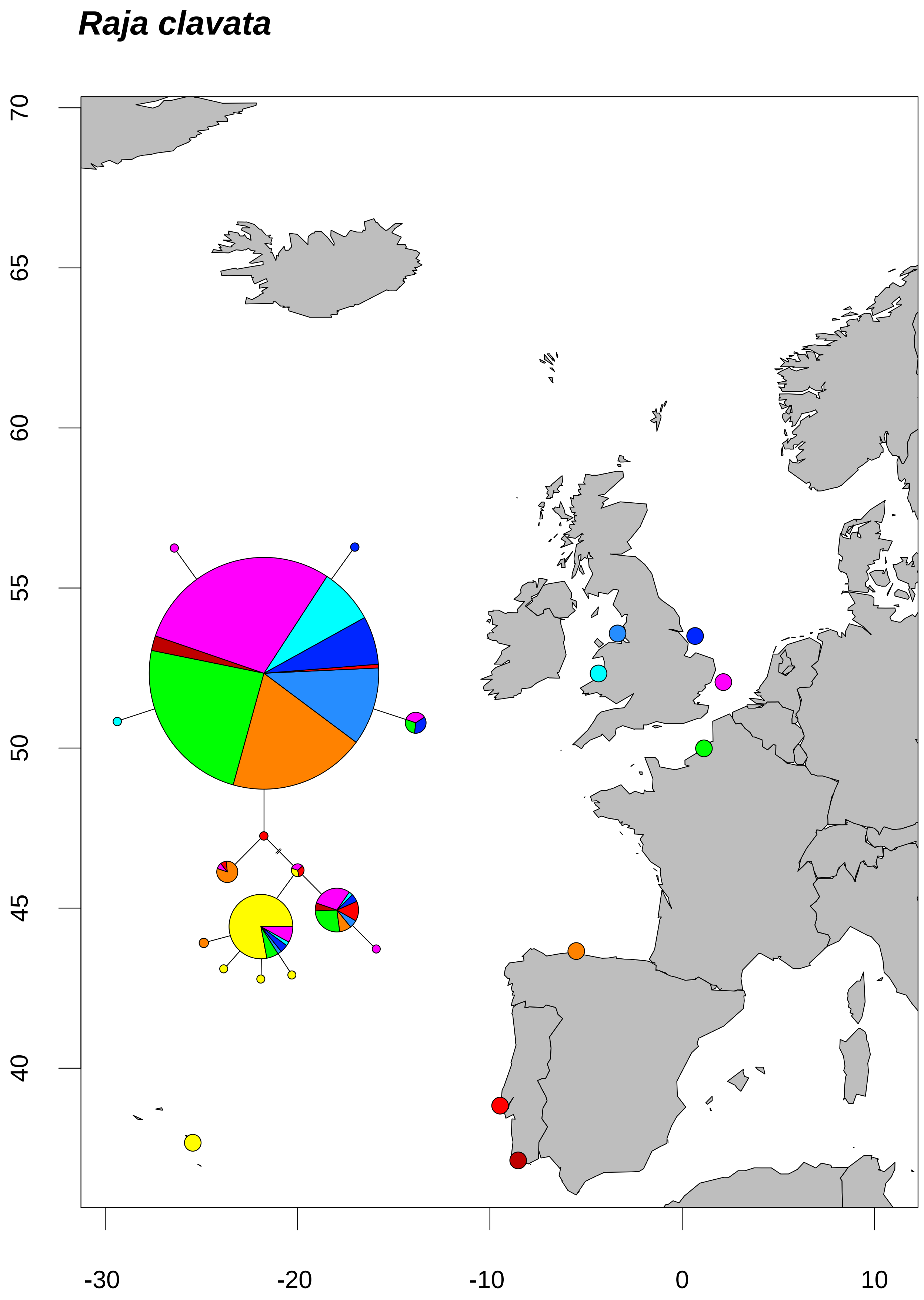

Figure 19

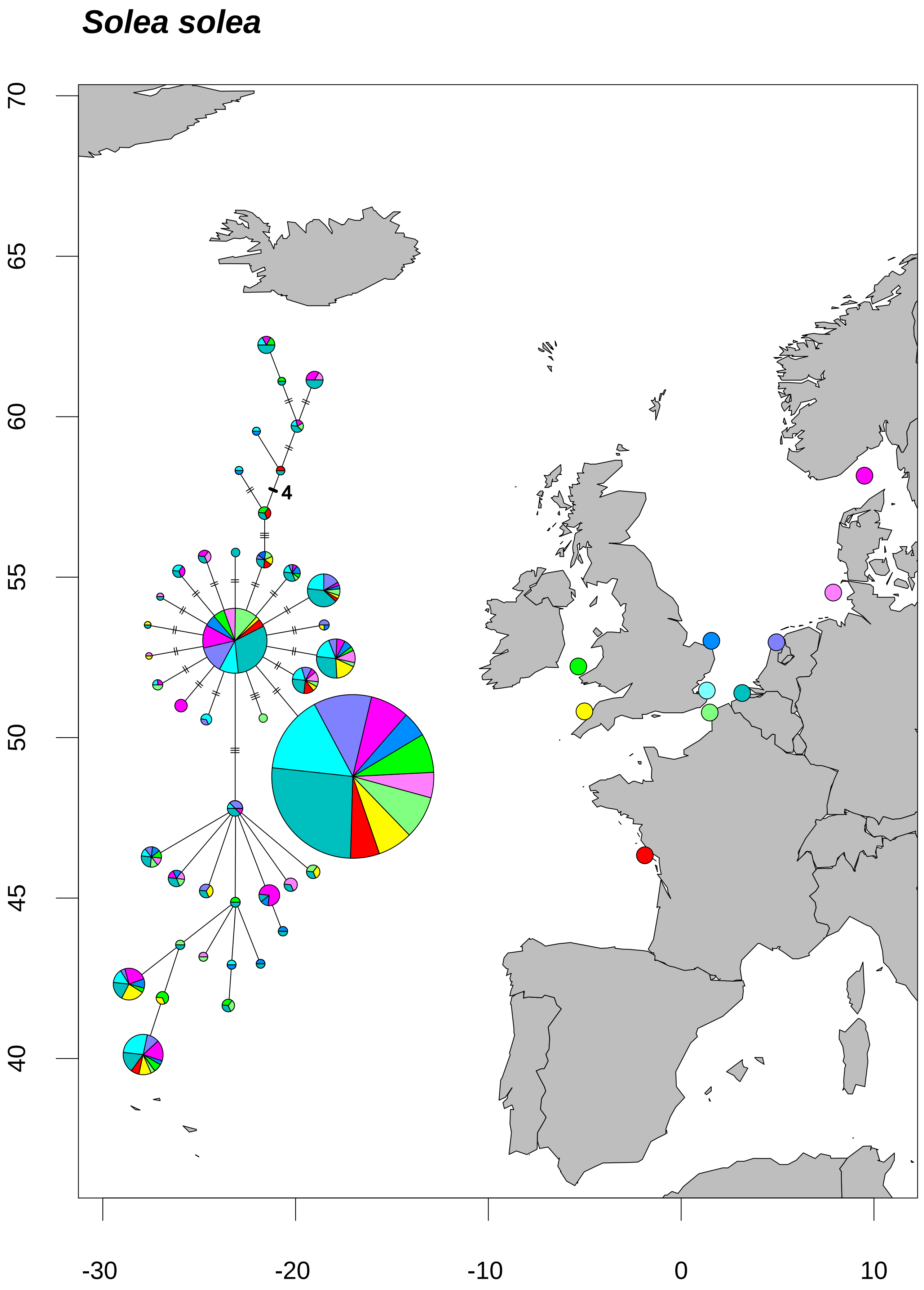

Figure 20

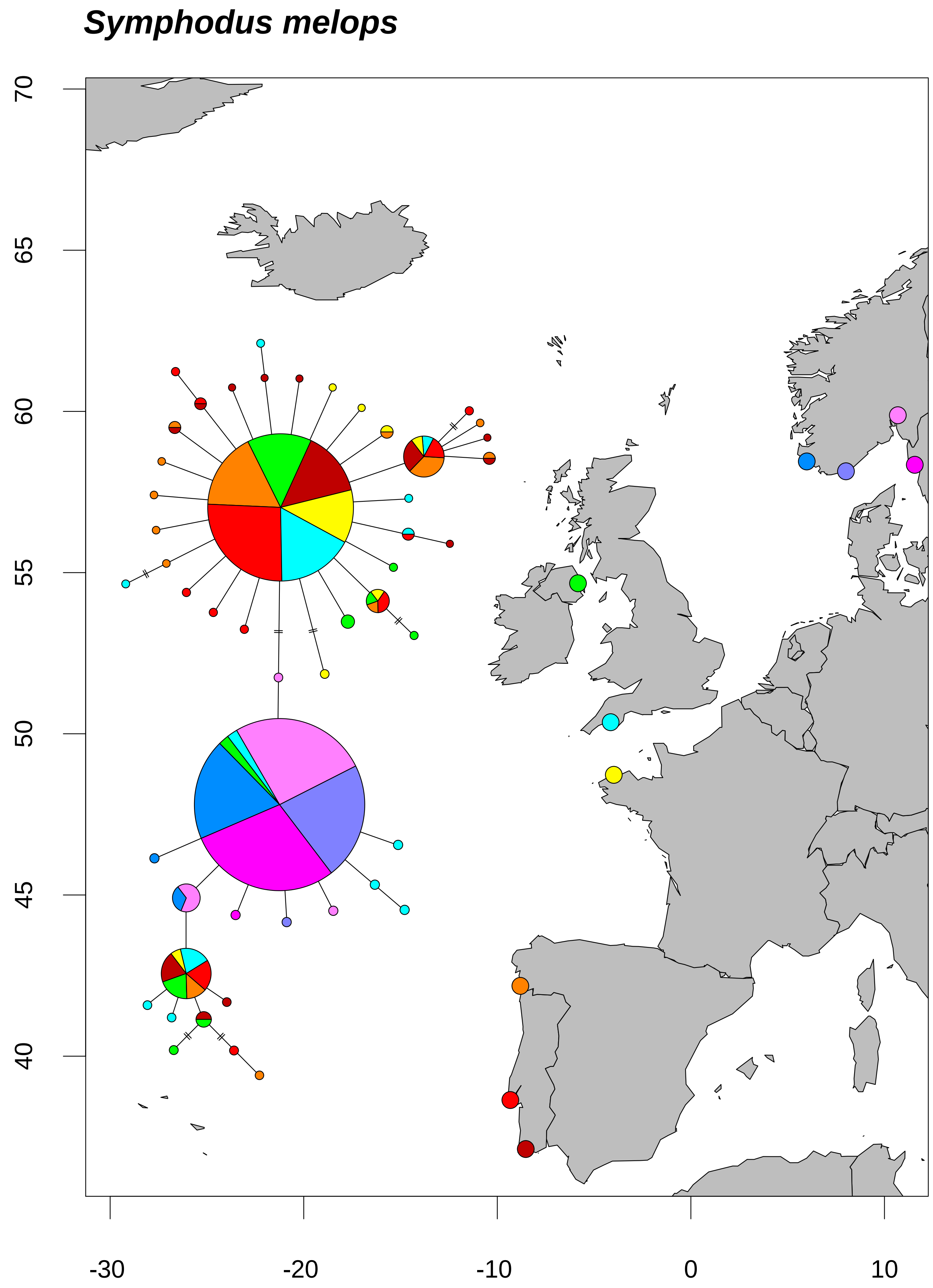

Figure 21
