# Supplementary material for: Meta-analysis of northeast Atlantic marine taxa shows contrasting phylogeographic patterns following post-LGM expansions
Source: PeerJ. 2018 Sep 28;6:e5684. doi: 10.7717/peerj.5684 (PMC6166638; doi:10.7717/peerj.5684)
Supplement: Figure S3 [file peerj-06-5684-s005.pdf]

Mismatch analysis graphs for all 21 species used in the meta-analysis
Figure S3, Supporting Information

Tom Jenkins

March 7, 2018

List of Figures

1 *Carcinus maenas* 1
2 *Celleporella hyalina* 2
3 *Cerastoderma edule* 3
4 *Conger conger* 4
5 *Dicentrarchus labrax* 5
6 *Labrus bergylta* 6
7 *Macoma balthica* 7
8 *Macoma balthica* lineage 1 8
9 *Macoma balthica* lineage 2 9
10 *Maja brachydactyla* 10
11 *Modiolus modiolus* 11
12 *Modiolus modiolus* lineage 1 12
13 *Nassarius nitidus* 13
14 *Nassarius reticulatus* 14
15 *Neomysis integer* 15
16 *Owenia fusiformis* 16
17 *Owenia fusiformis* lineage 1 17
18 *Owenia fusiformis* lineage 2 18

19 *Owenia fusiformis* lineage 3 . . . 19
20 *Pomatoschistus microps* . . . 20
21 *Pomatoschistus minutus* . . . 21
22 *Palinurus elephas* . . . 22
23 *Pectinaria koreni* . . . 23
24 *Pectinaria koreni* lineage 1 . . . 24
25 *Pectinaria koreni* lineage 2 . . . 25
26 *Pelvetia canaliculata* . . . 26
27 *Raja clavata* . . . 27
28 *Solea solea* . . . 28
29 *Symphodus melops* . . . 29

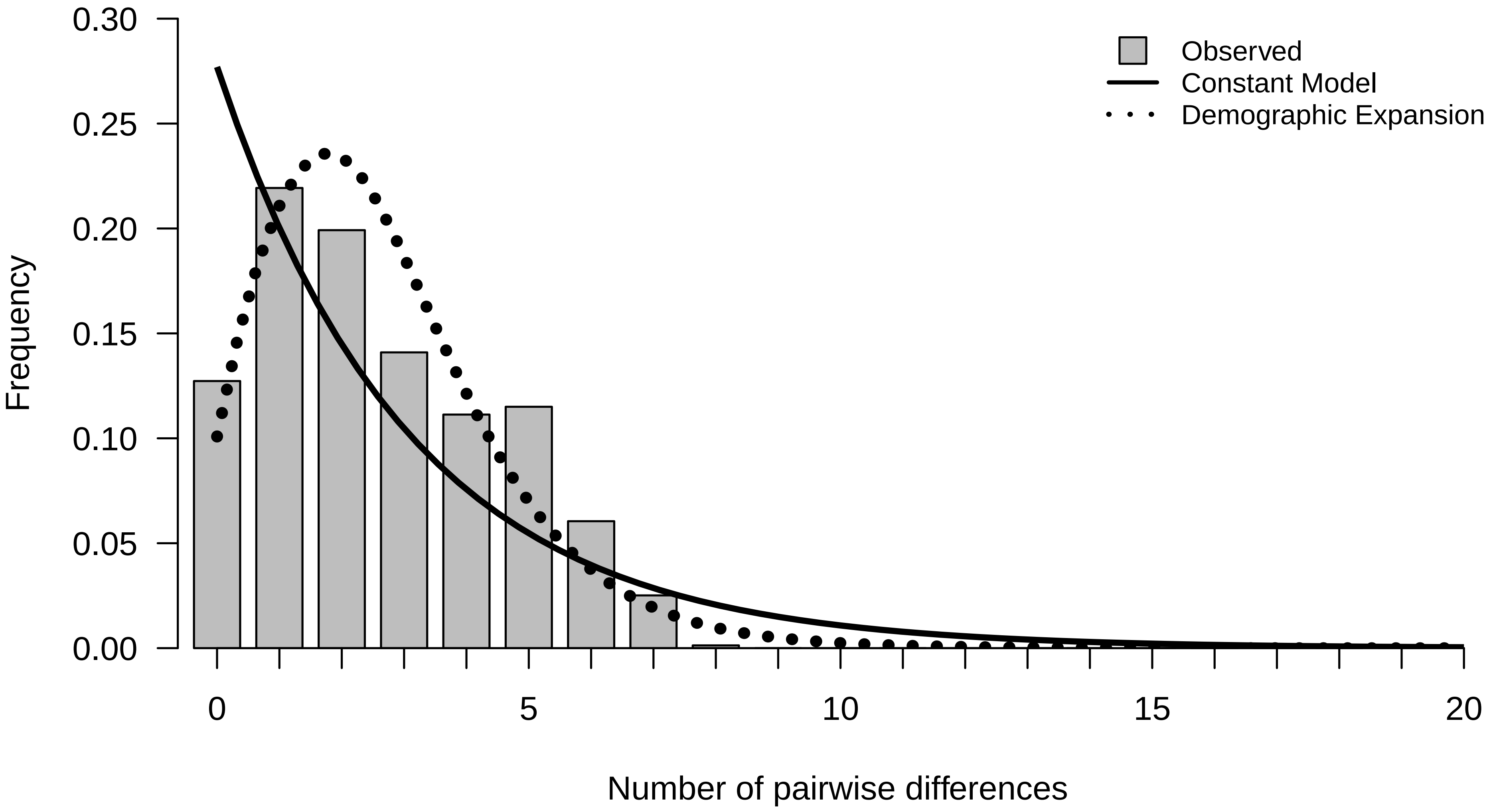

Figure 1: *Carcinus maenas* mismatch analysis

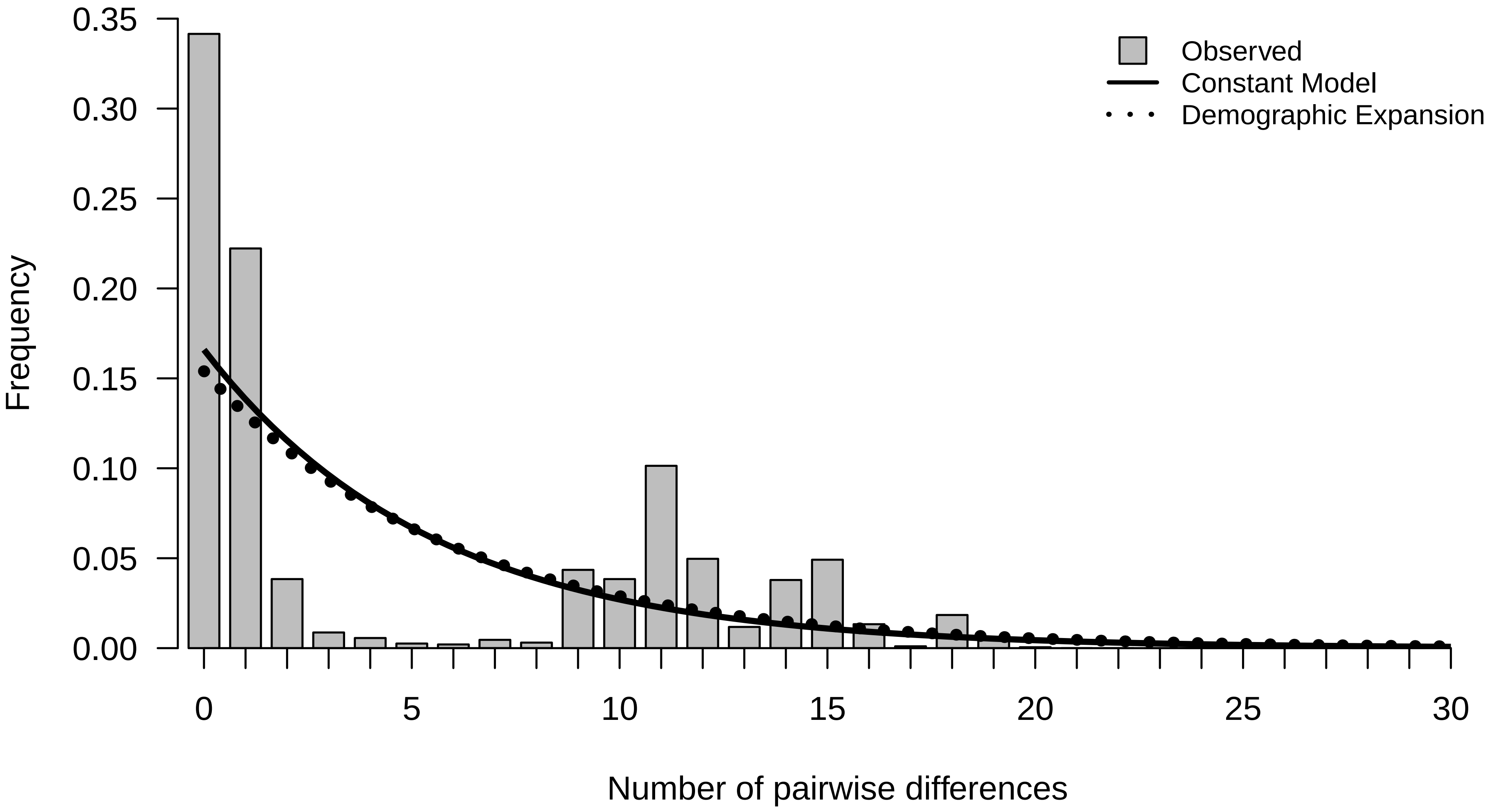

Figure 2: *Celleporella hyalina* mismatch analysis

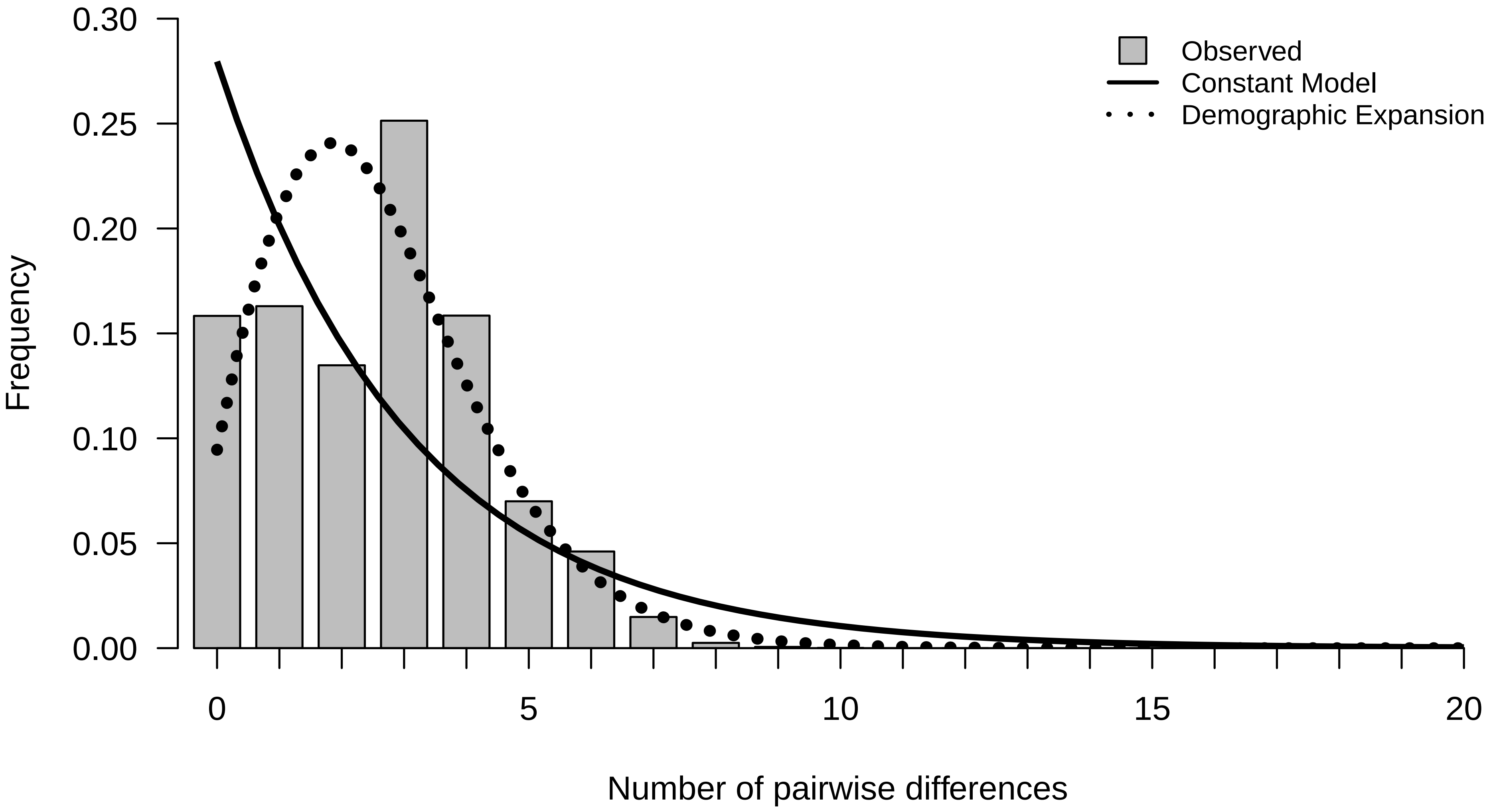

Figure 3: *Cerastoderma edule* mismatch analysis

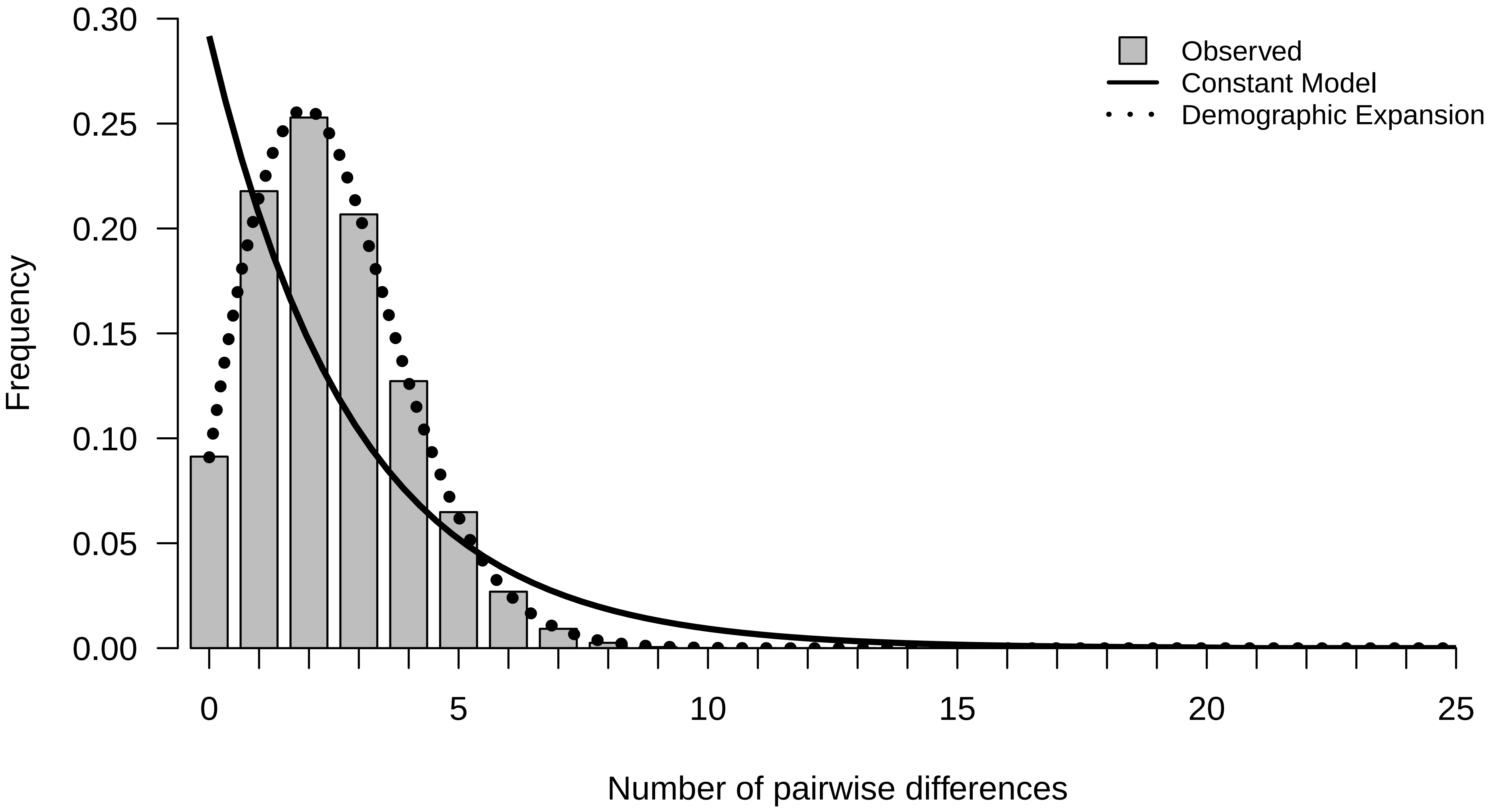

Figure 4: *Conger conger* mismatch analysis

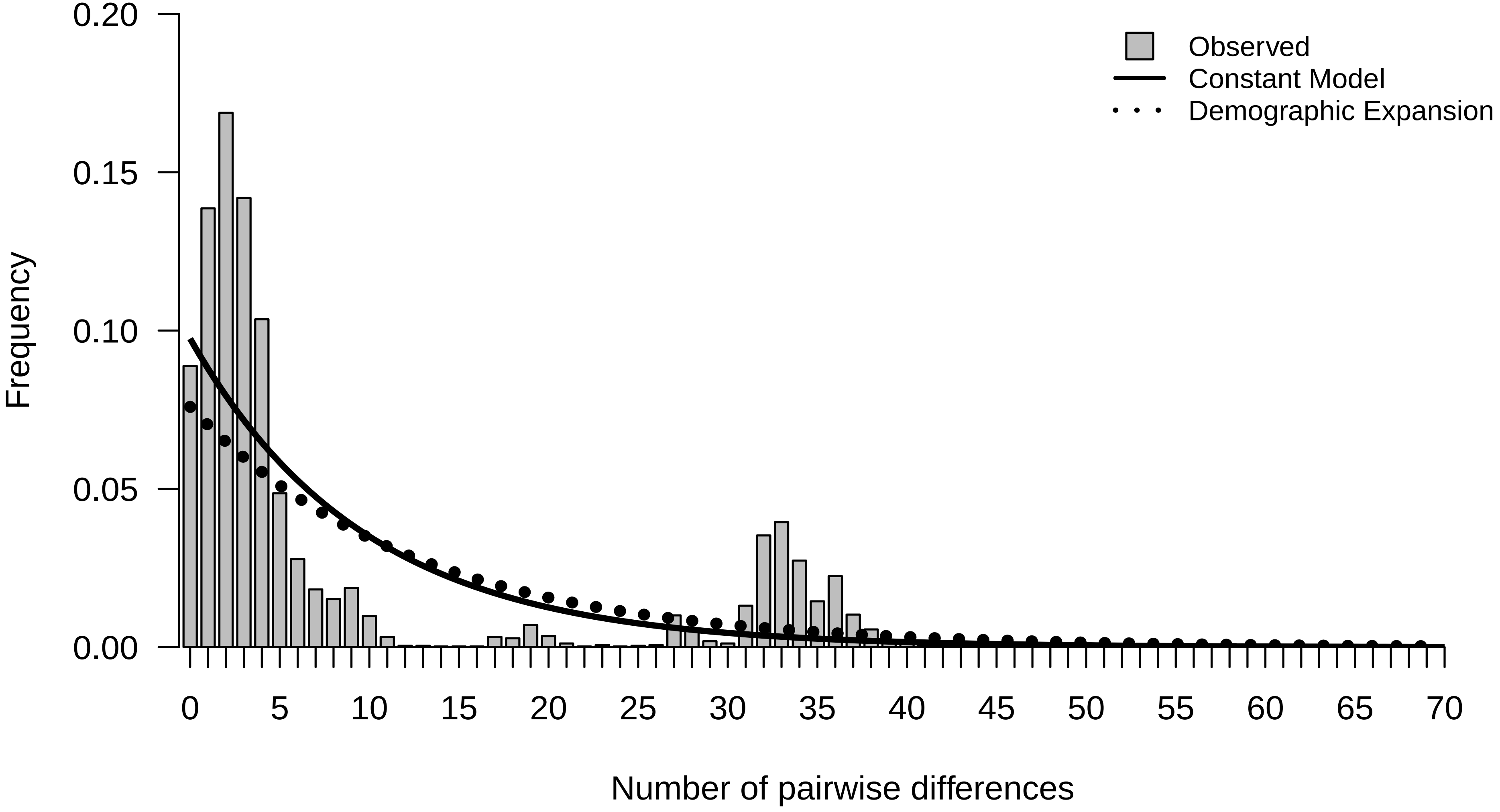

Figure 5: *Dicentrarchus labrax* mismatch analysis

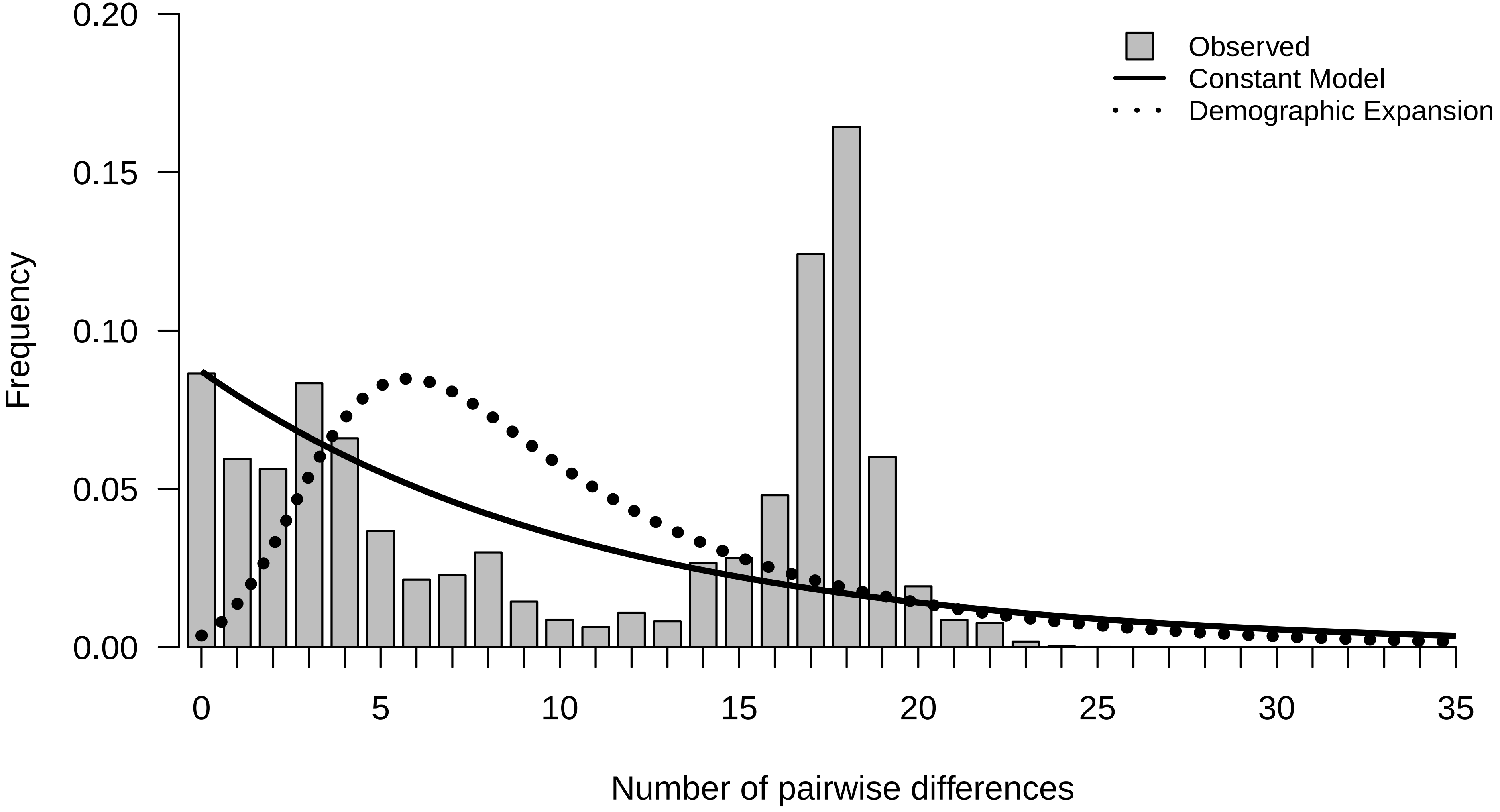

Figure 6: *Labrus bergylta* mismatch analysis

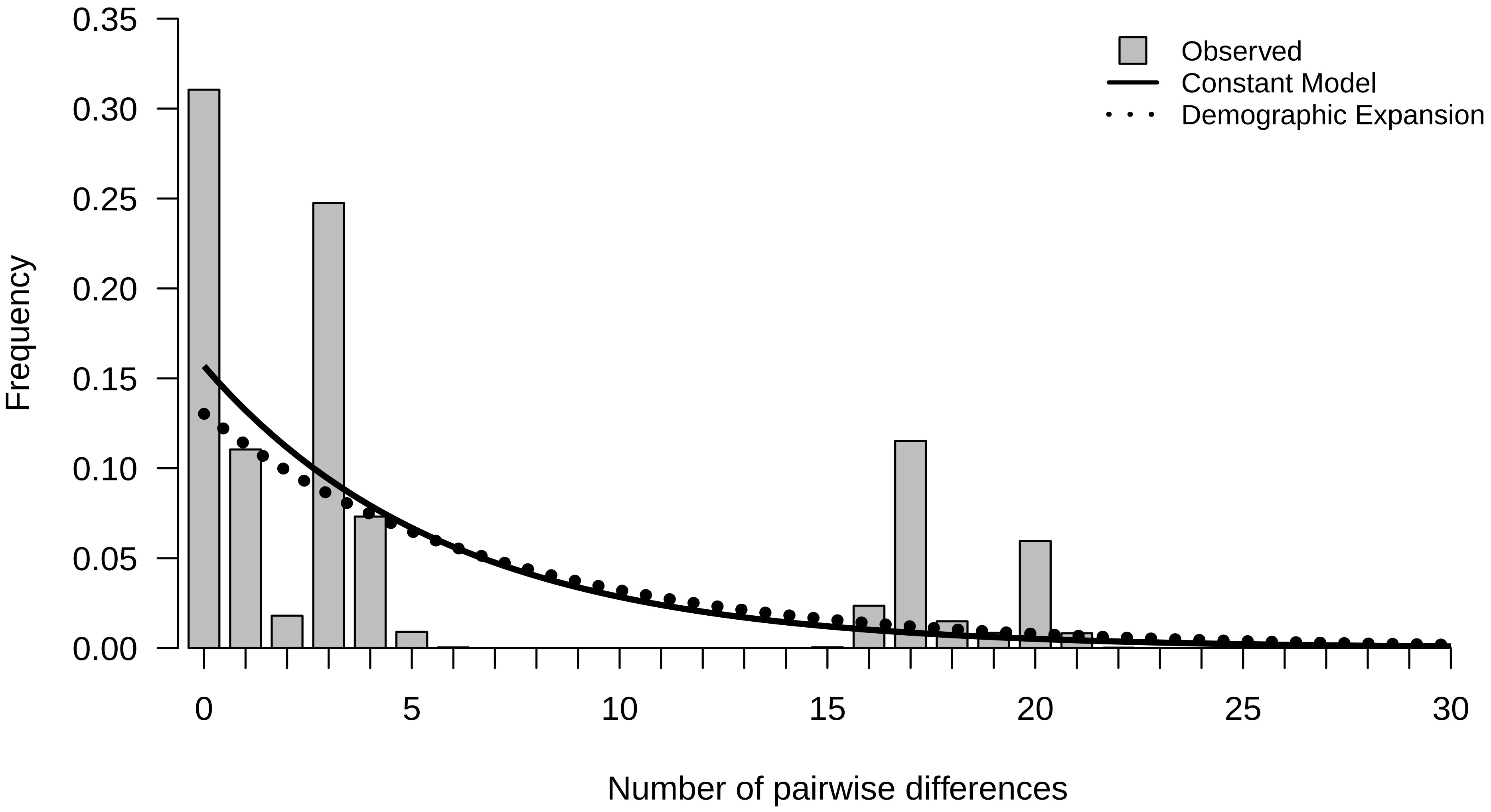

Figure 7: *Macoma balthica* mismatch analysis

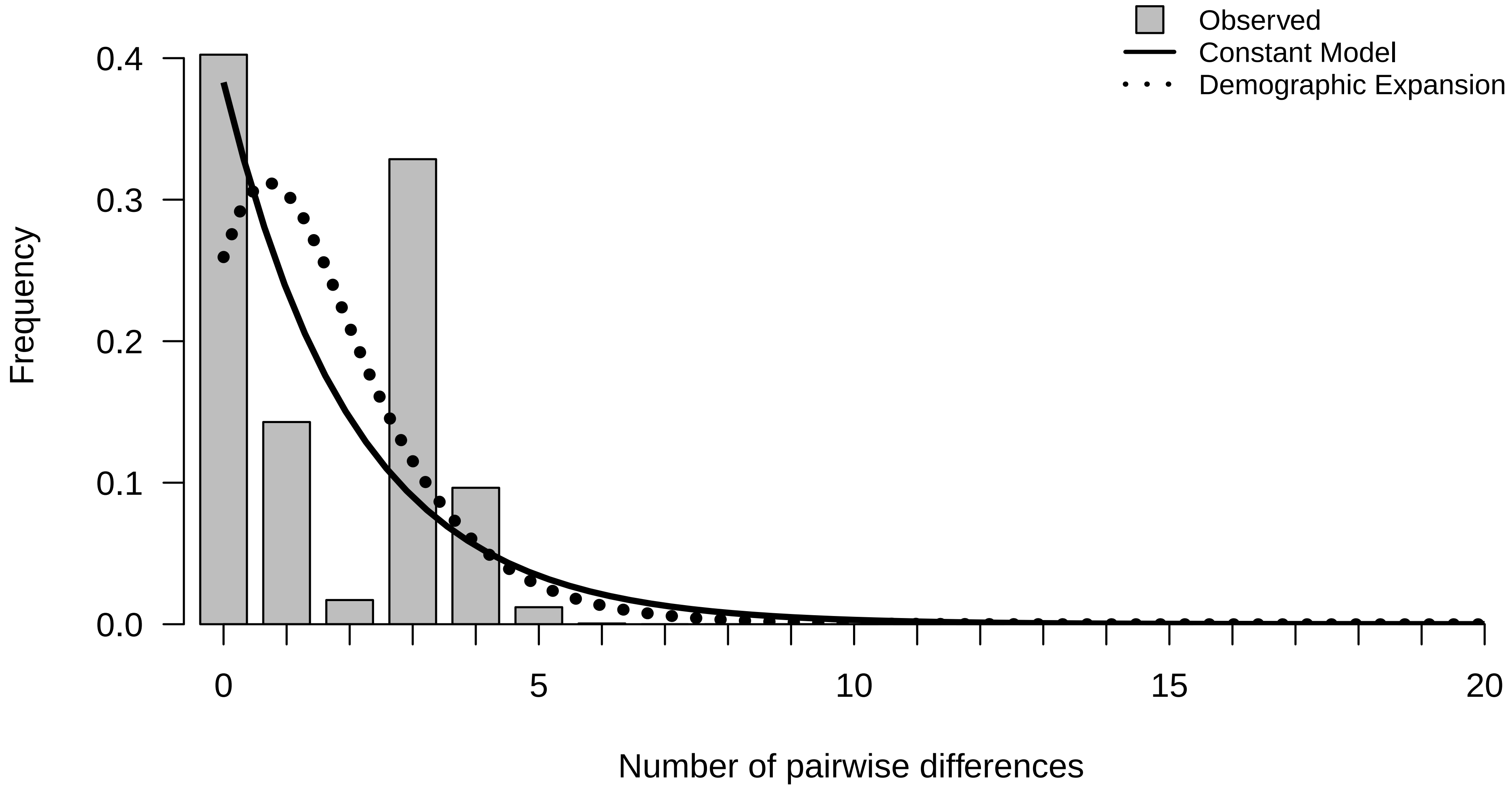

Figure 8: *Macoma balthica* lineage 1 mismatch analysis

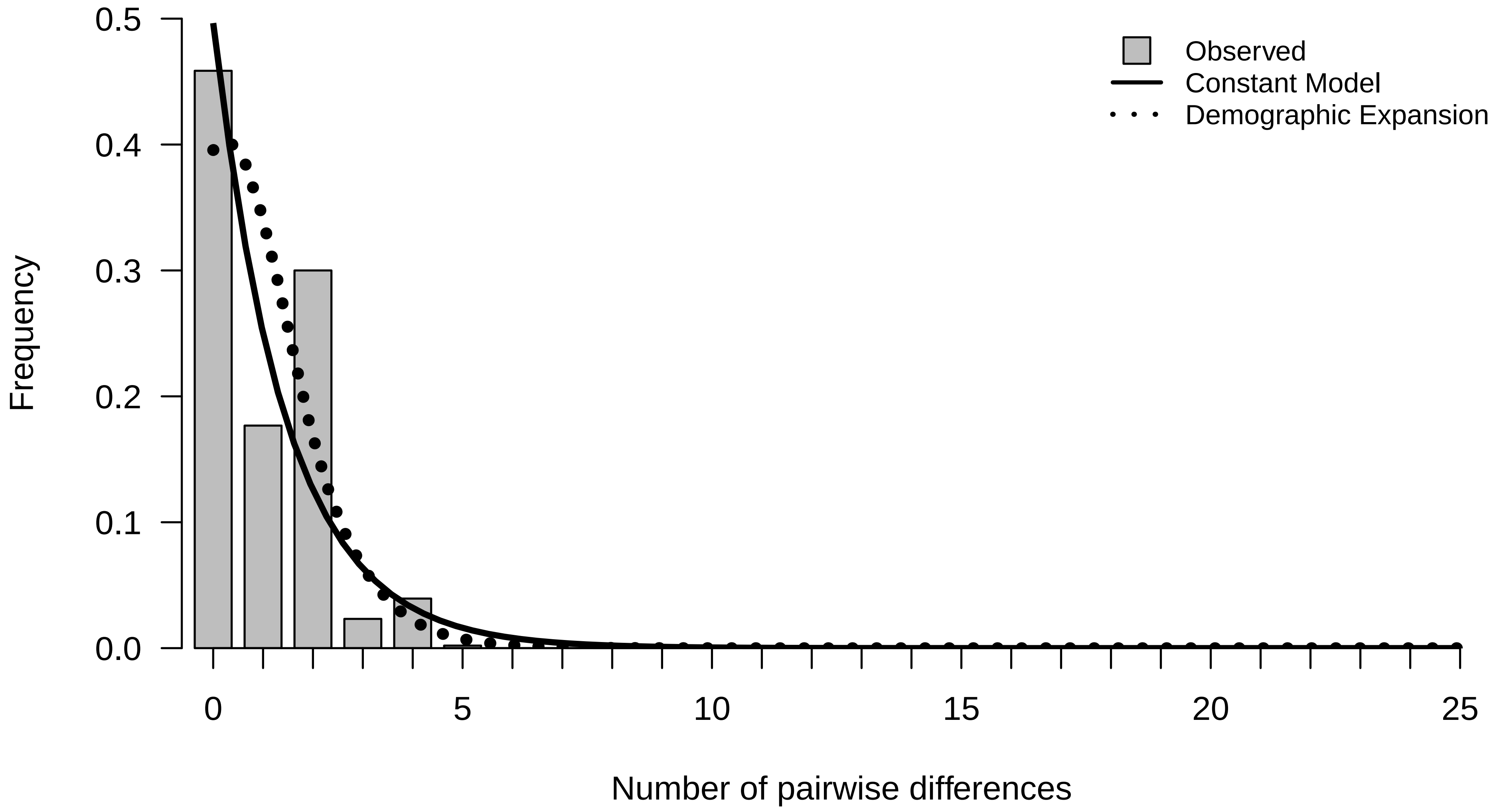

Figure 9: *Macoma balthica* lineage 2 mismatch analysis

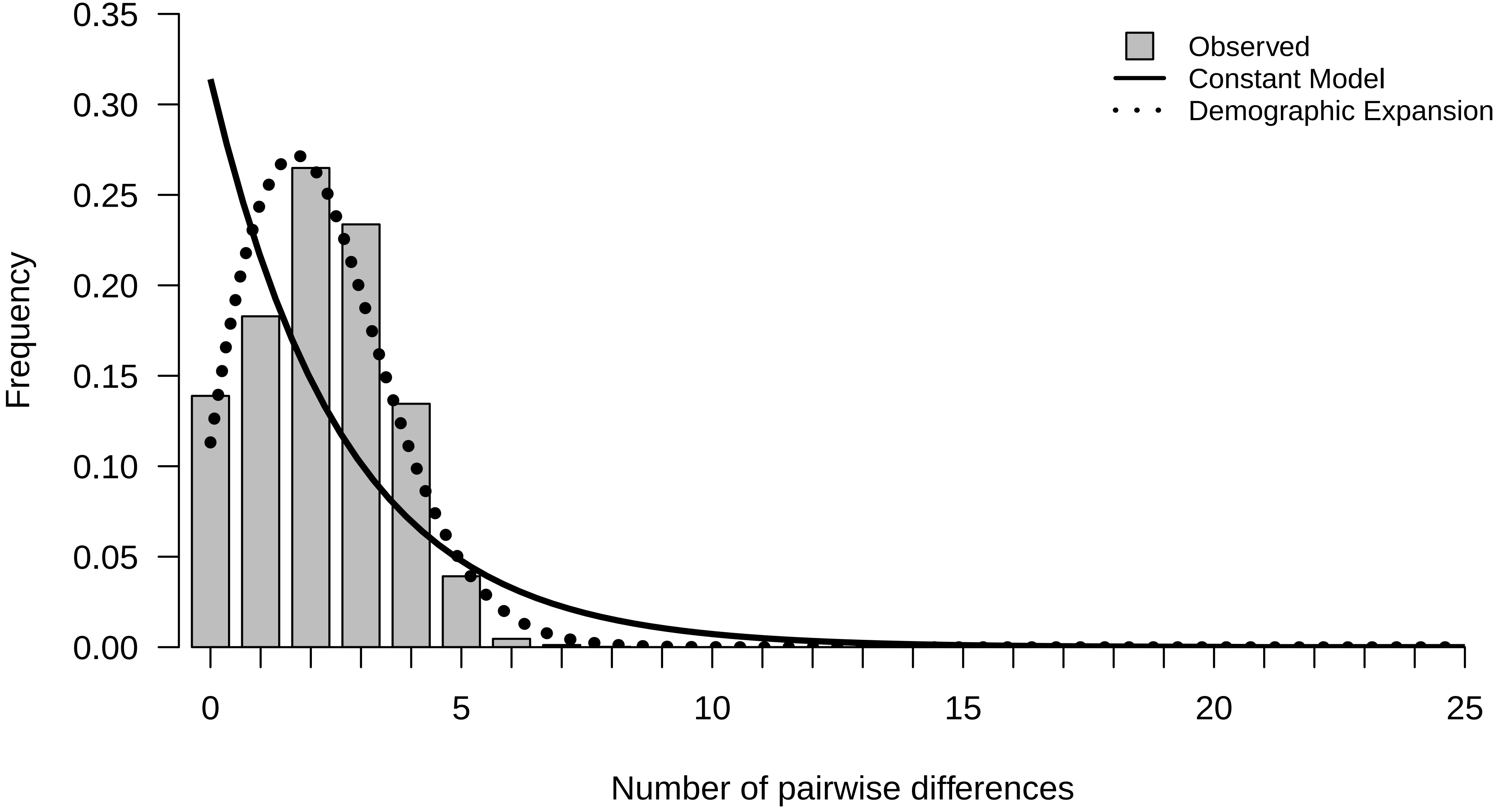

Figure 10: *Maja brachydactyla* mismatch analysis

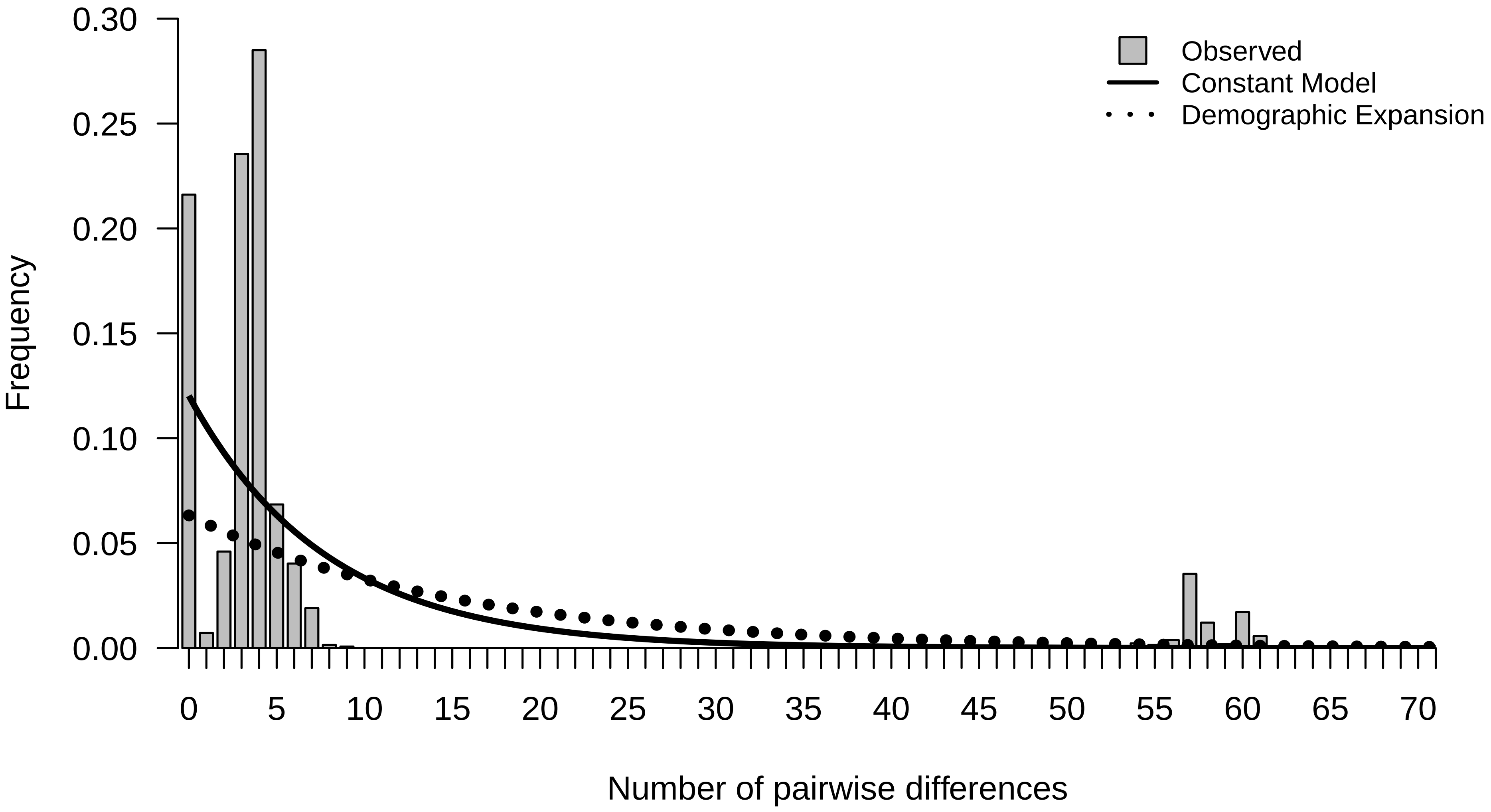

Figure 11: *Modiolus modiolus* mismatch analysis

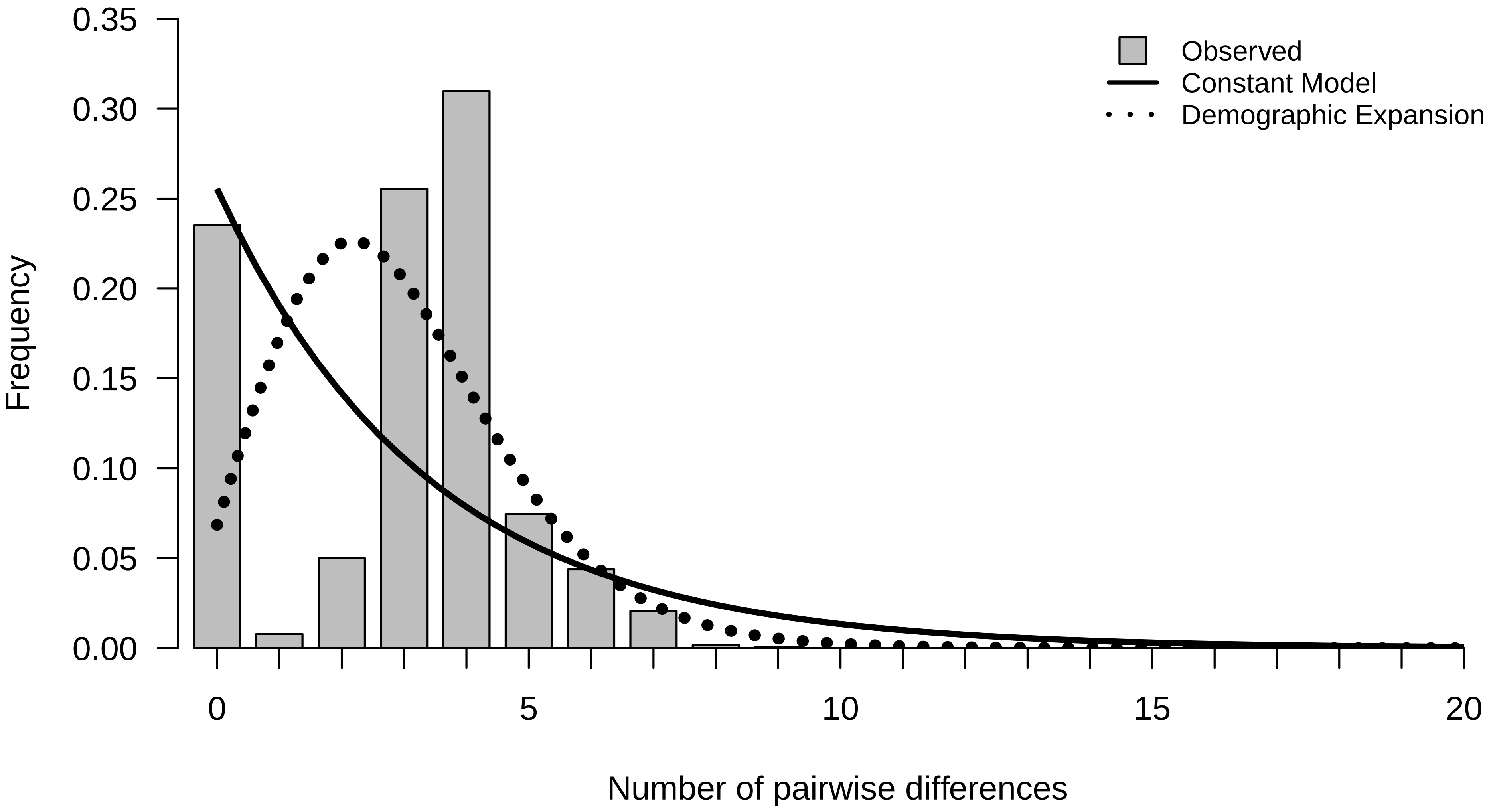

Figure 12: *Modiolus modiolus* lineage 1 mismatch analysis

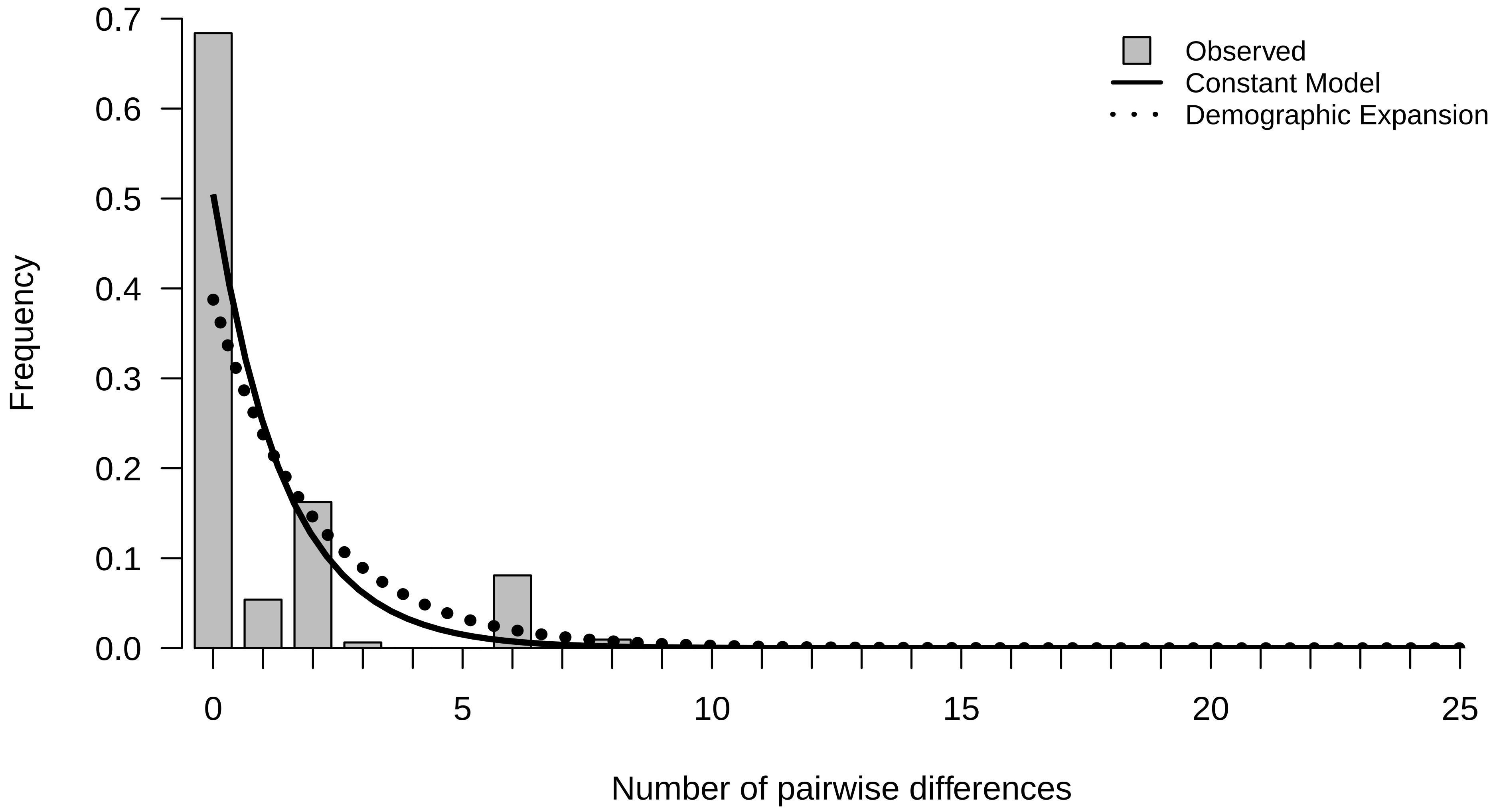

Figure 13: *Nassarius nitidus* mismatch analysis

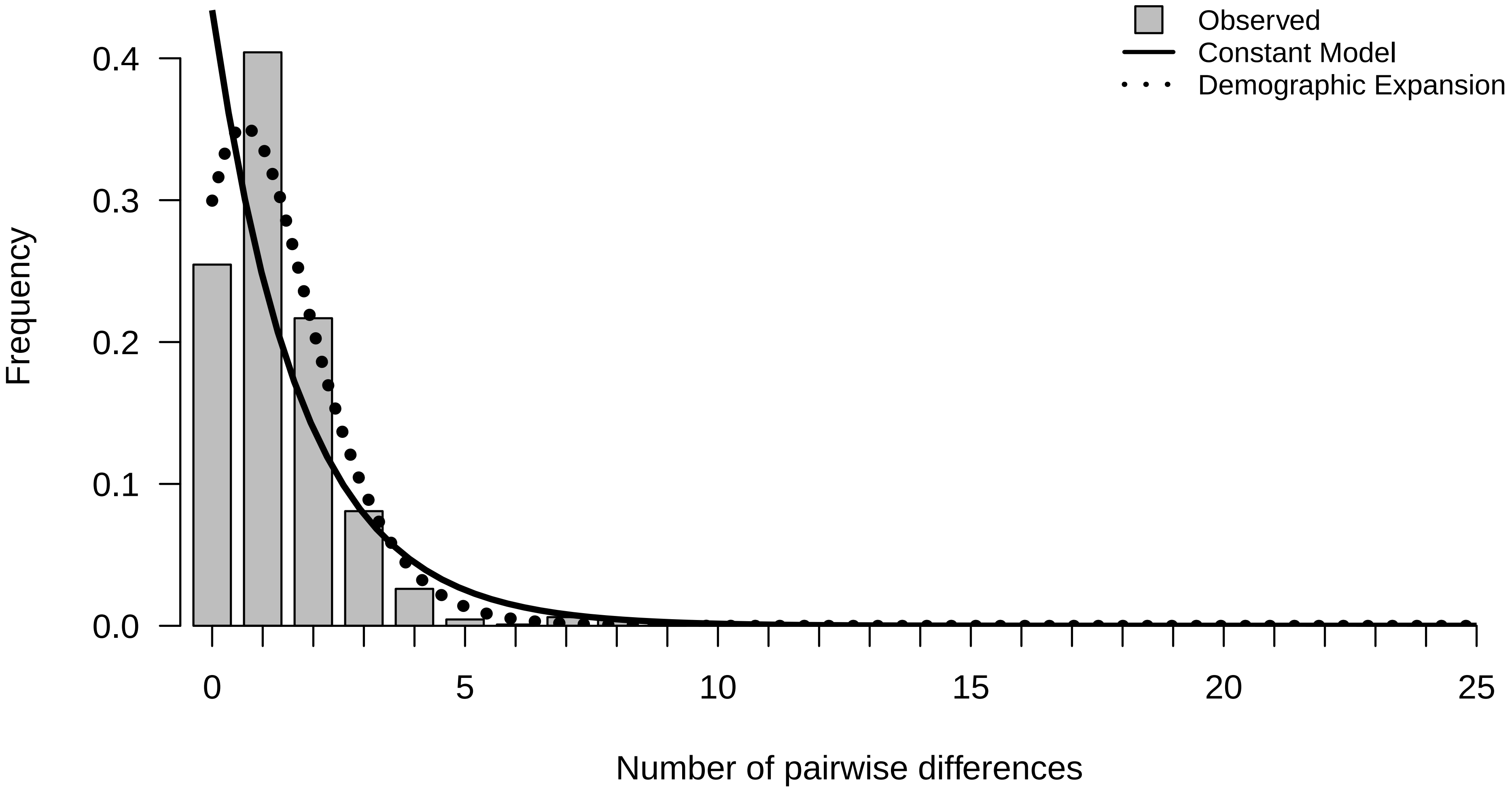

Figure 14: *Nassarius reticulatus* mismatch analysis

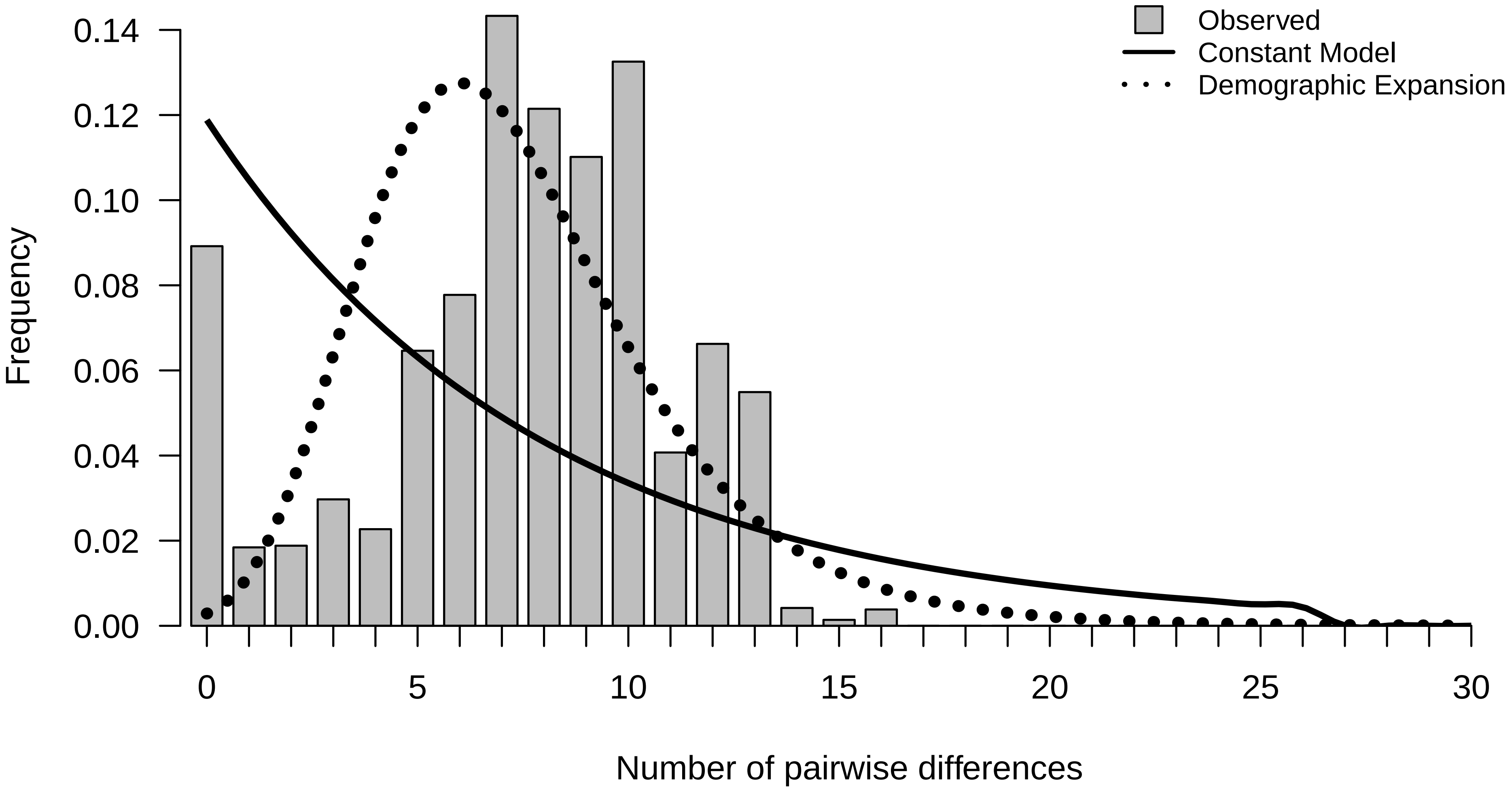

Figure 15: *Neomysis integer* mismatch analysis

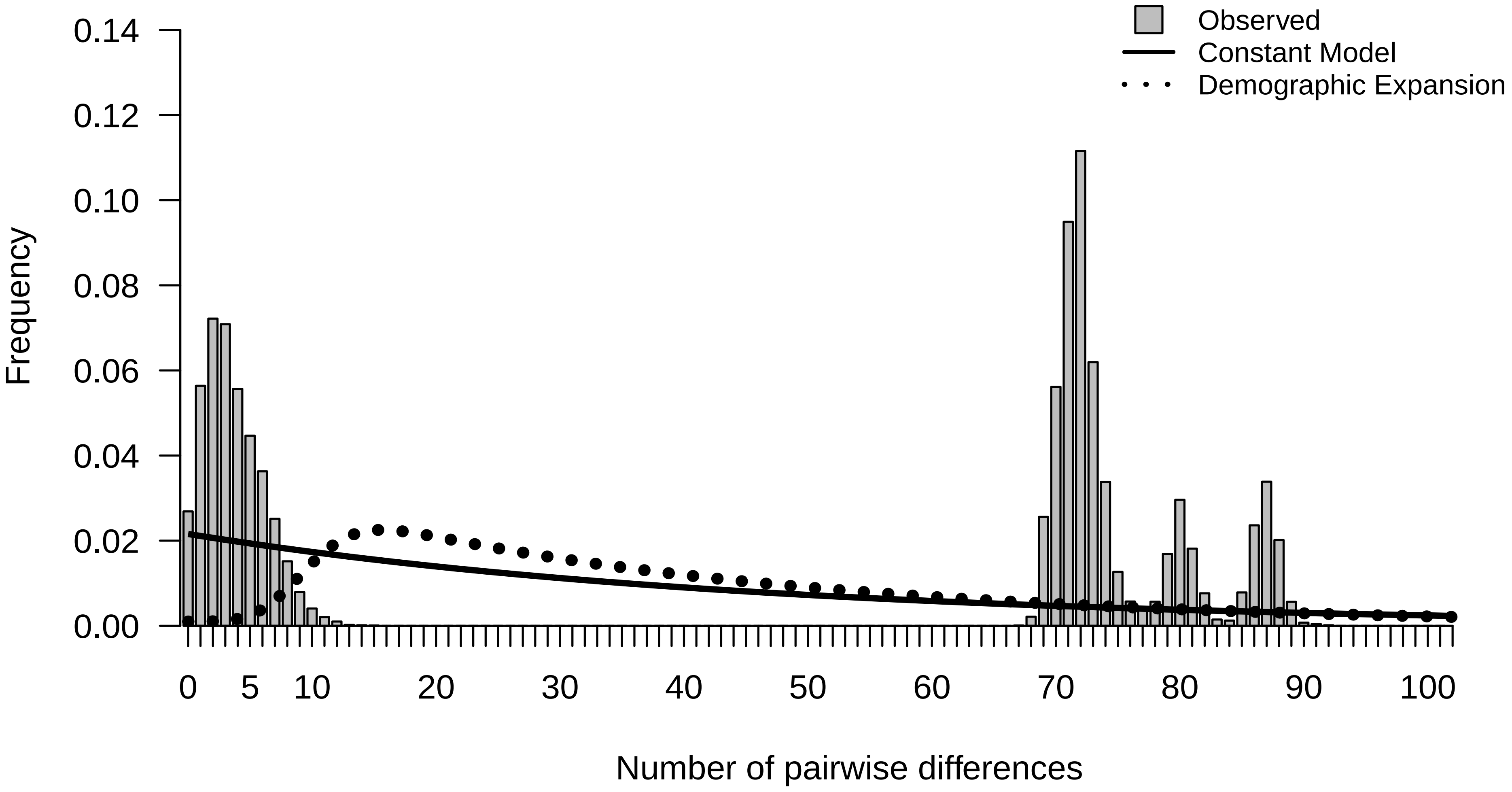

Figure 16: *Owenia fusiformis* mismatch analysis

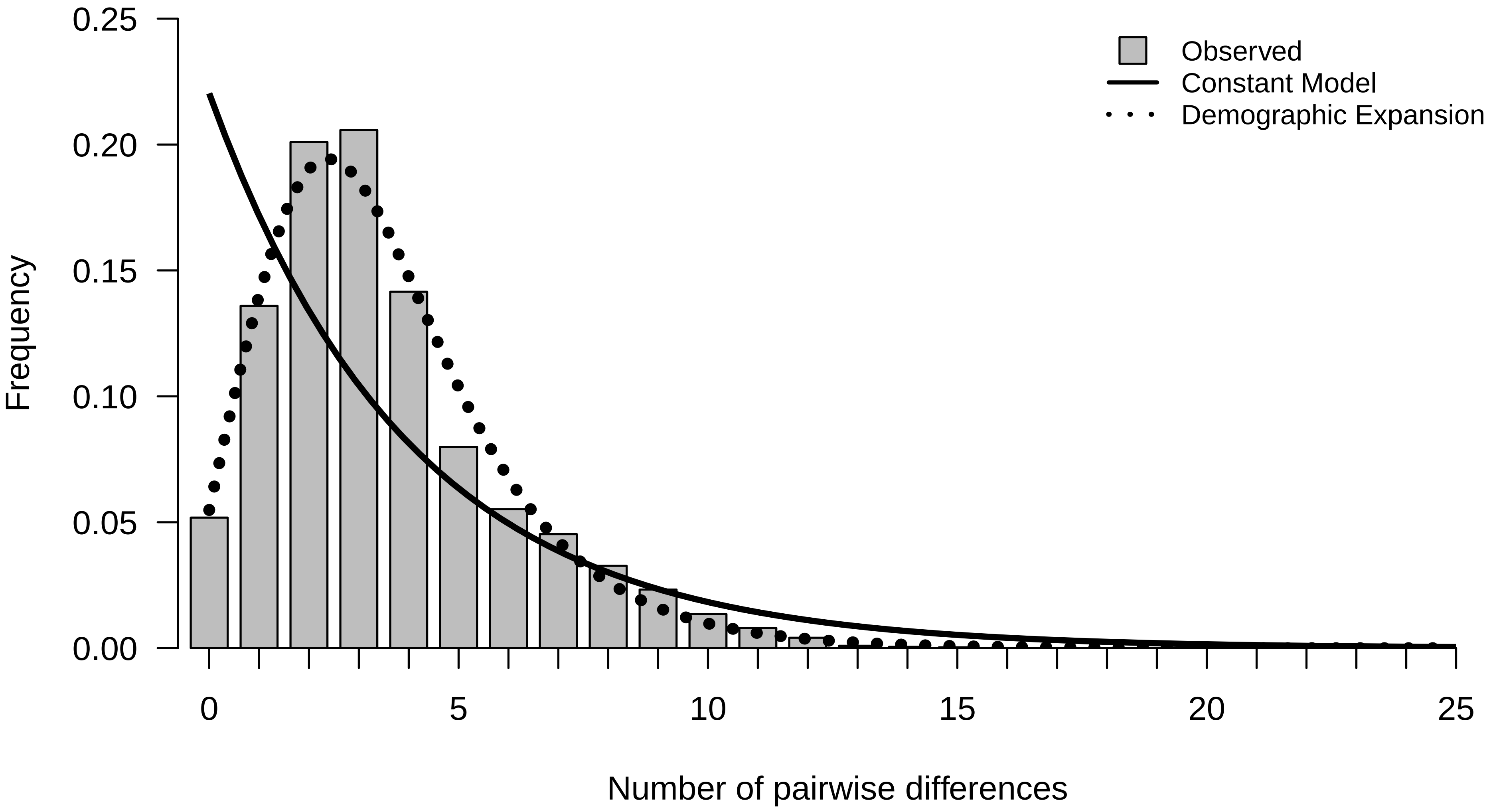

Figure 17: *Owenia fusiformis* lineage 1 mismatch analysis

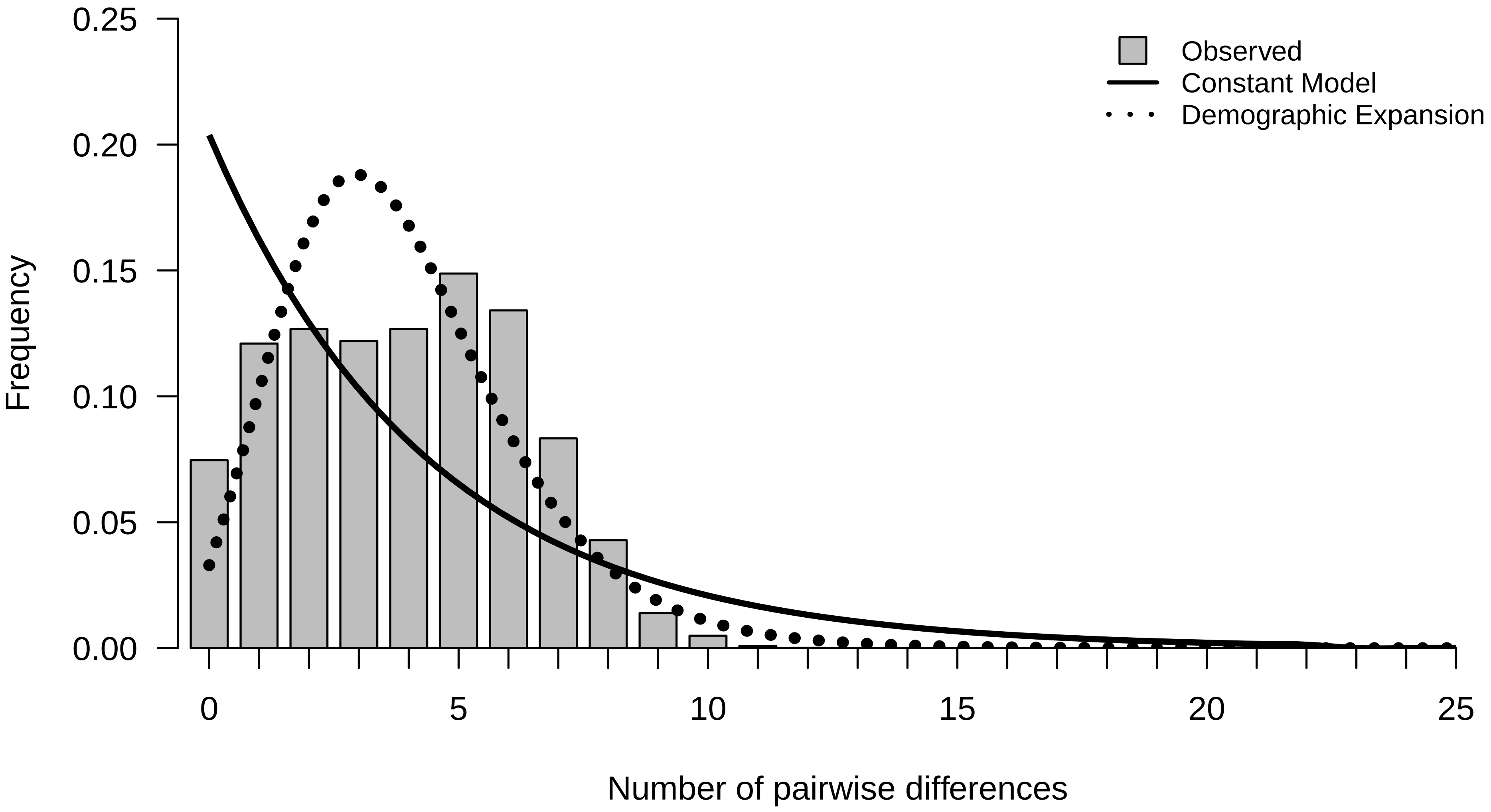

Figure 18: *Owenia fusiformis* lineage 2 mismatch analysis

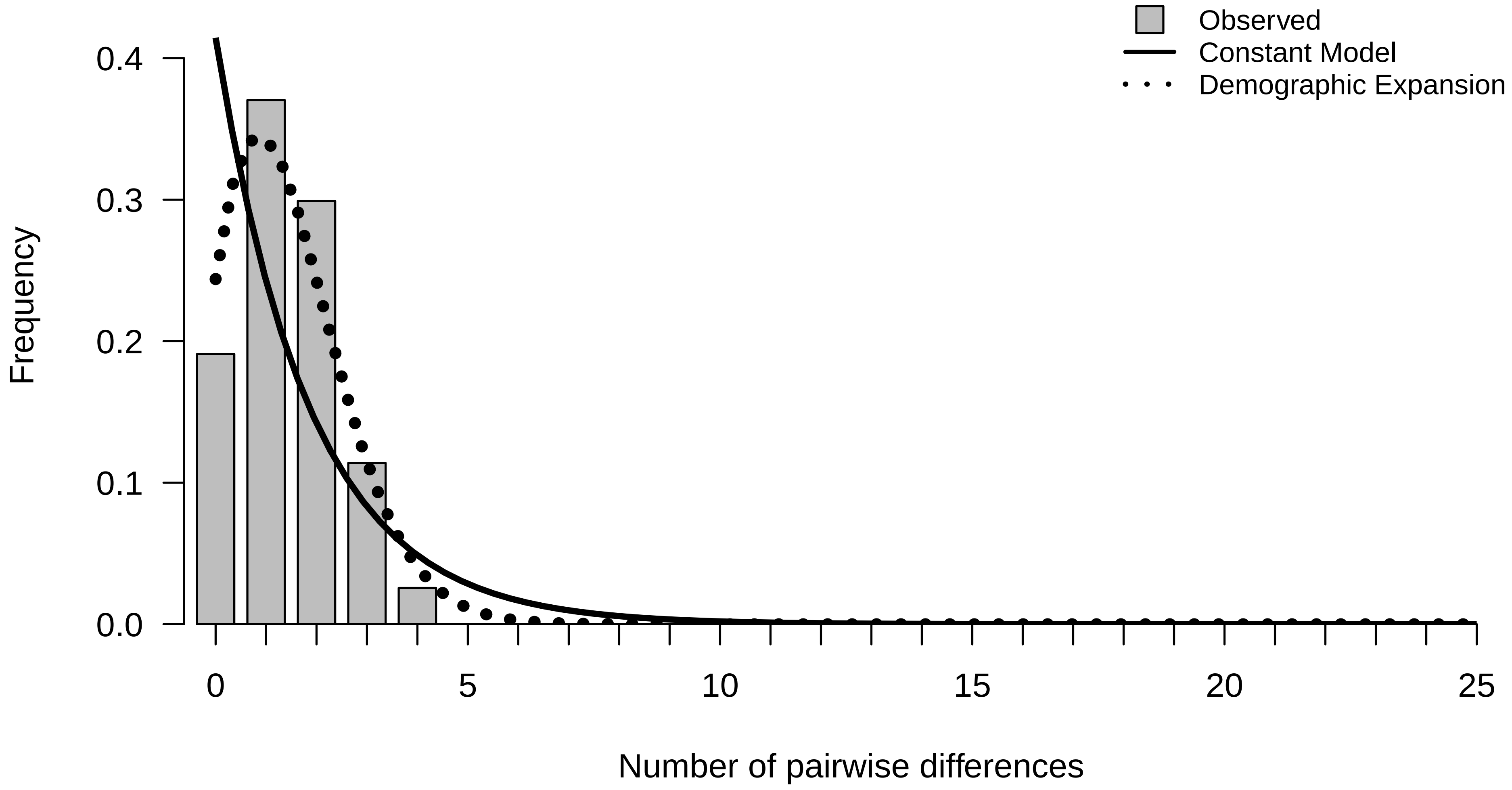

Figure 19: *Owenia fusiformis* lineage 3 mismatch analysis

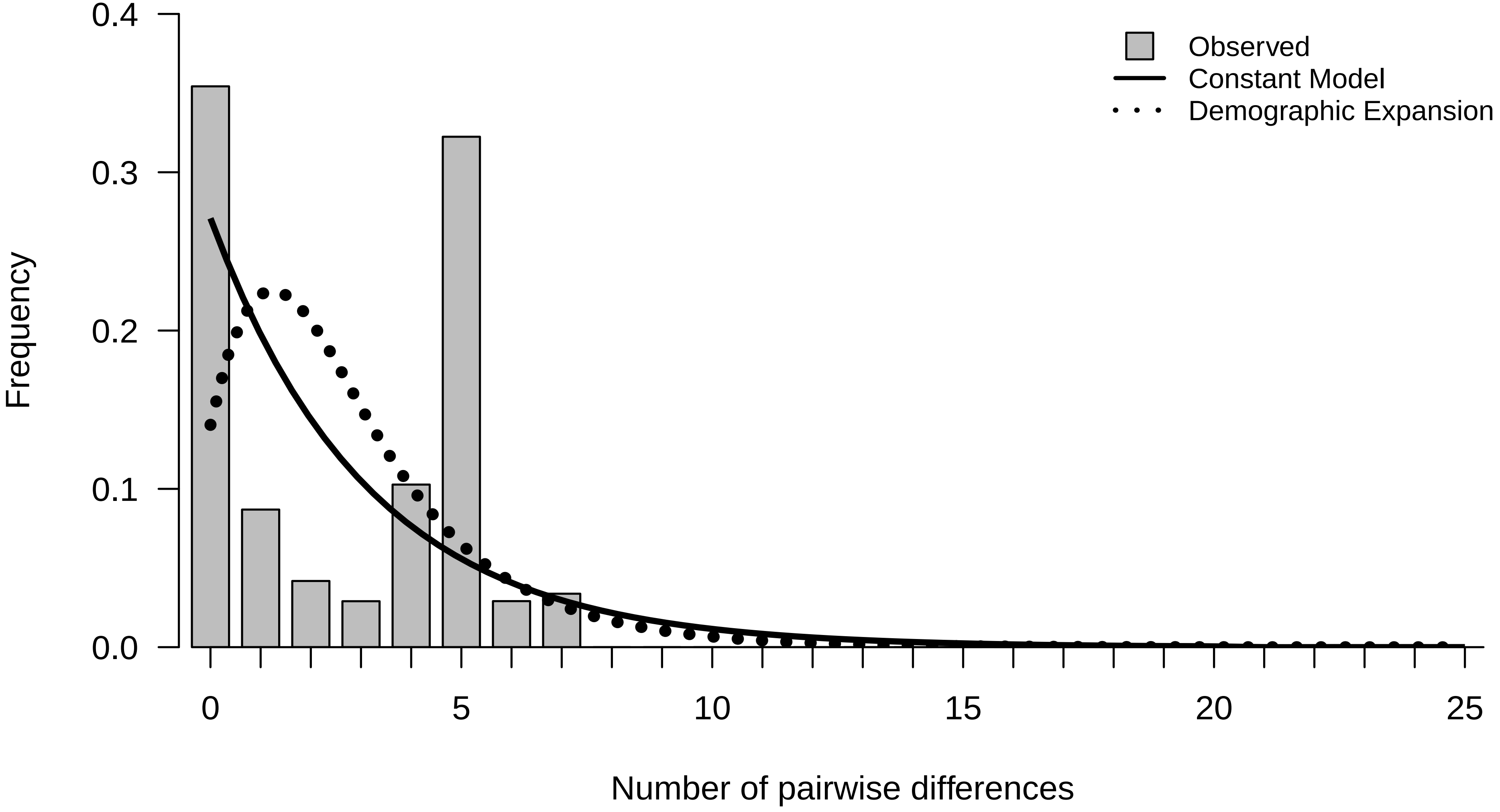

Figure 20: *Pomatoschistus microps* mismatch analysis

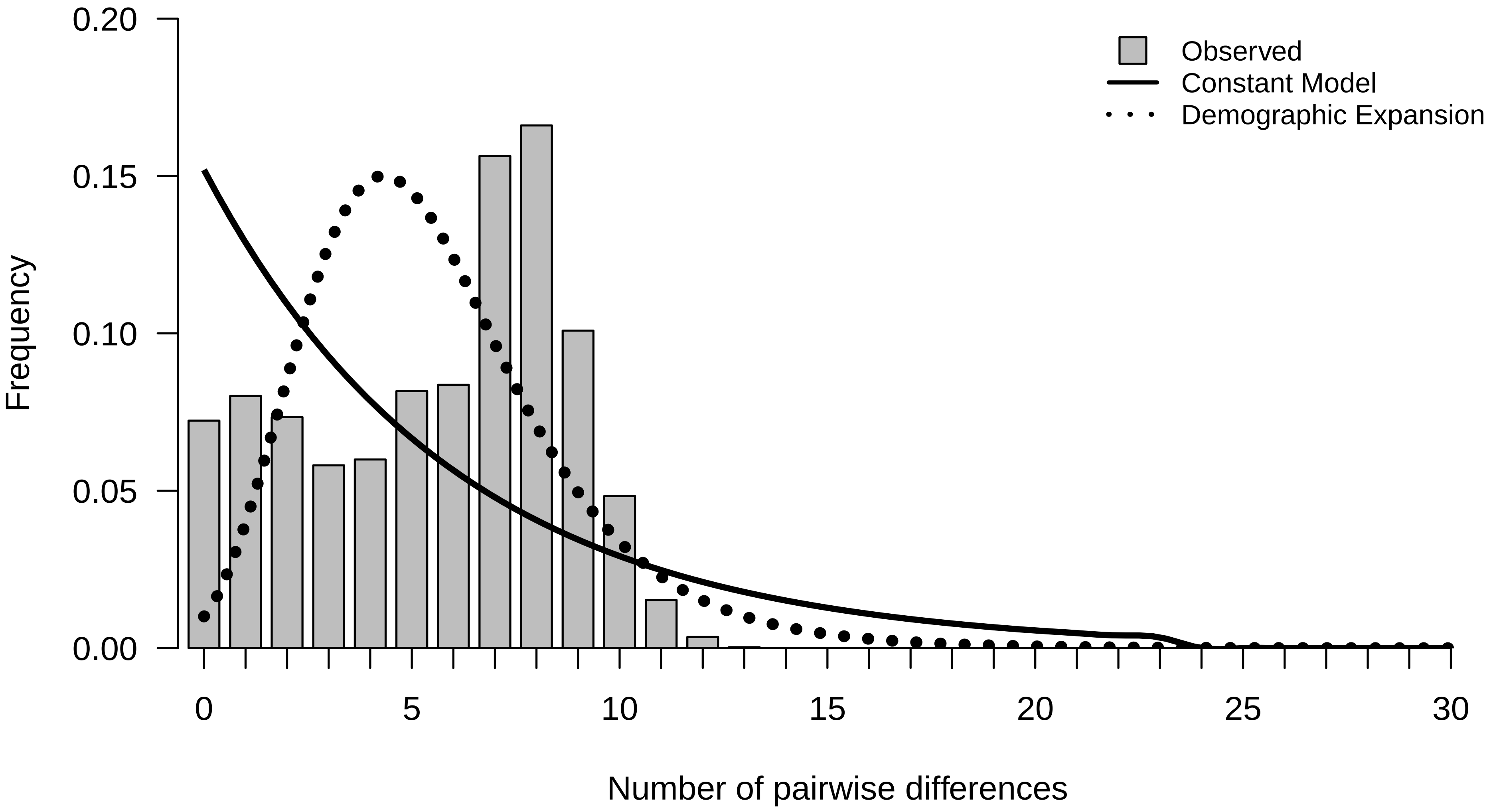

Figure 21: *Pomatoschistus minutus* mismatch analysis

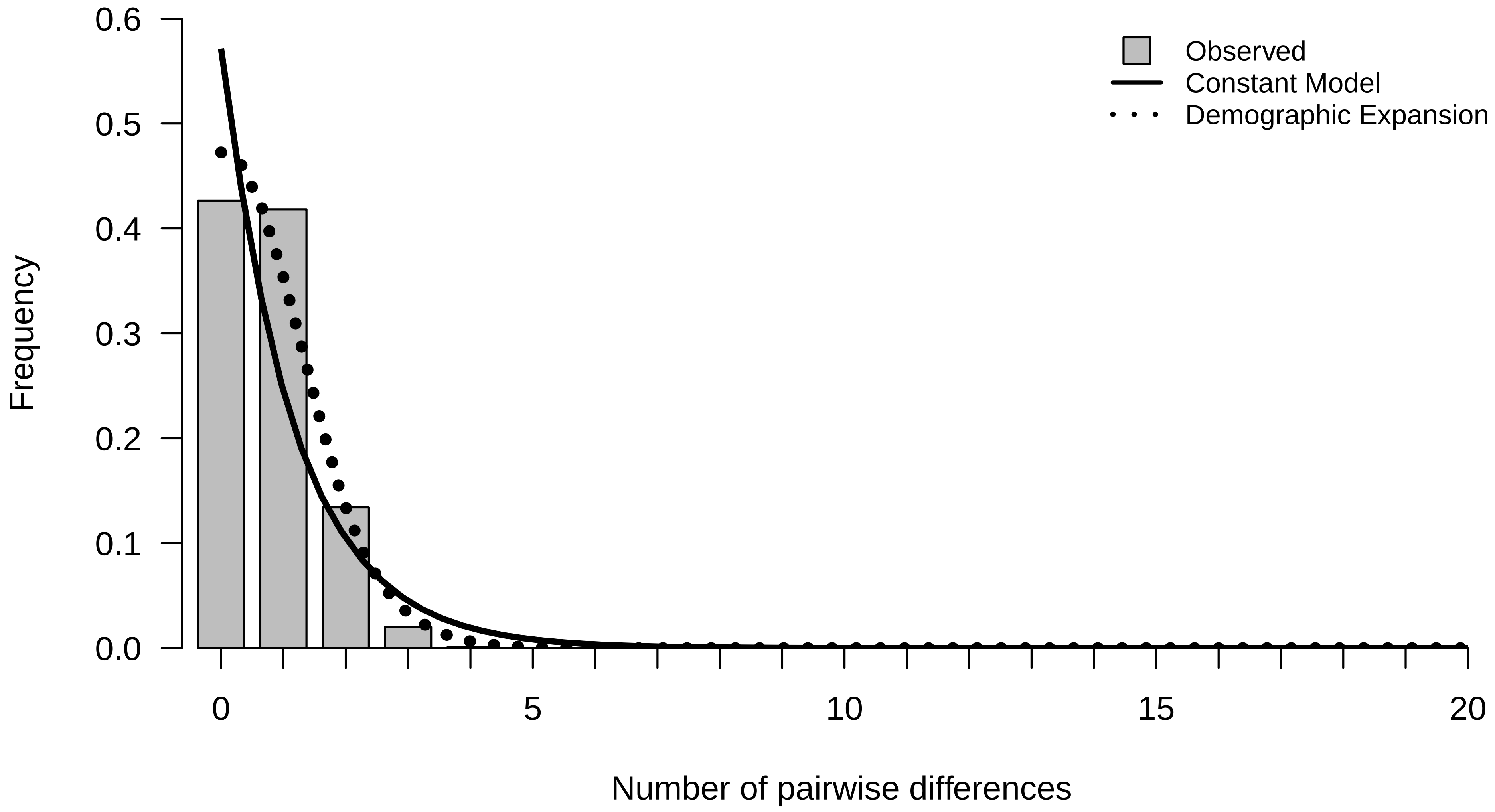

Figure 22: *Palinurus elephas* mismatch analysis

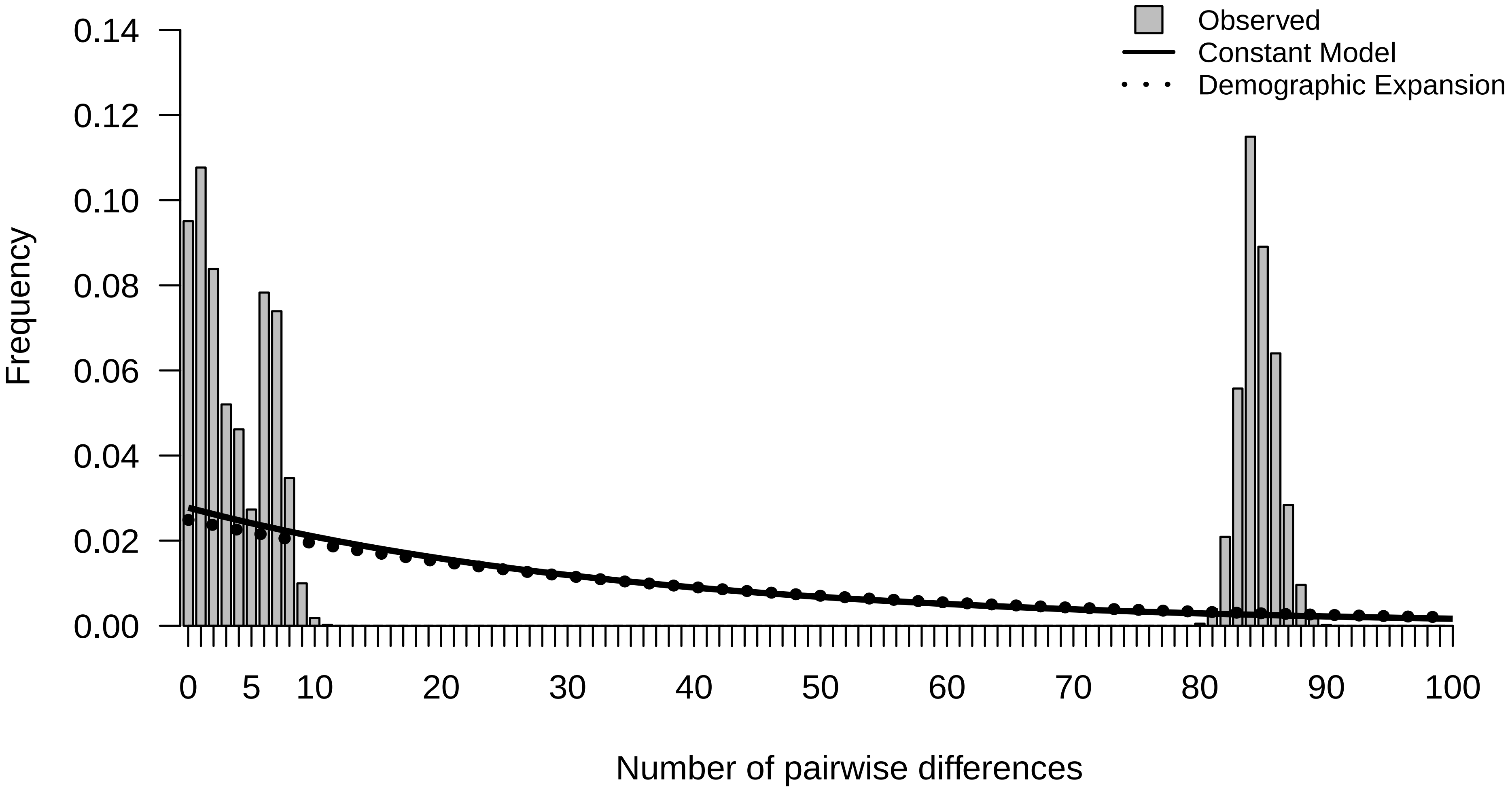

Figure 23: *Pectinaria koreni* mismatch analysis

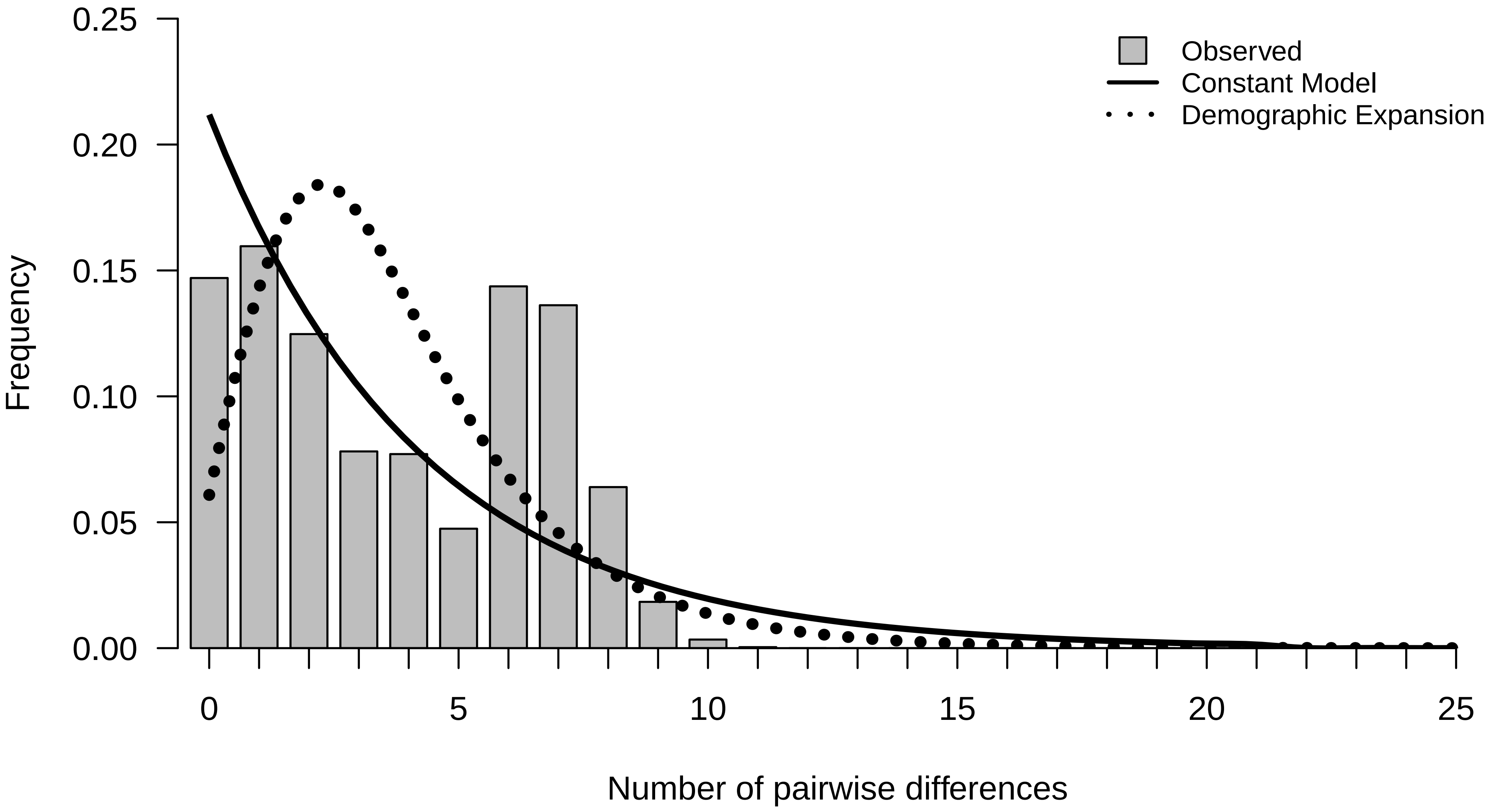

Figure 24: *Pectinaria koreni* lineage 1 mismatch analysis

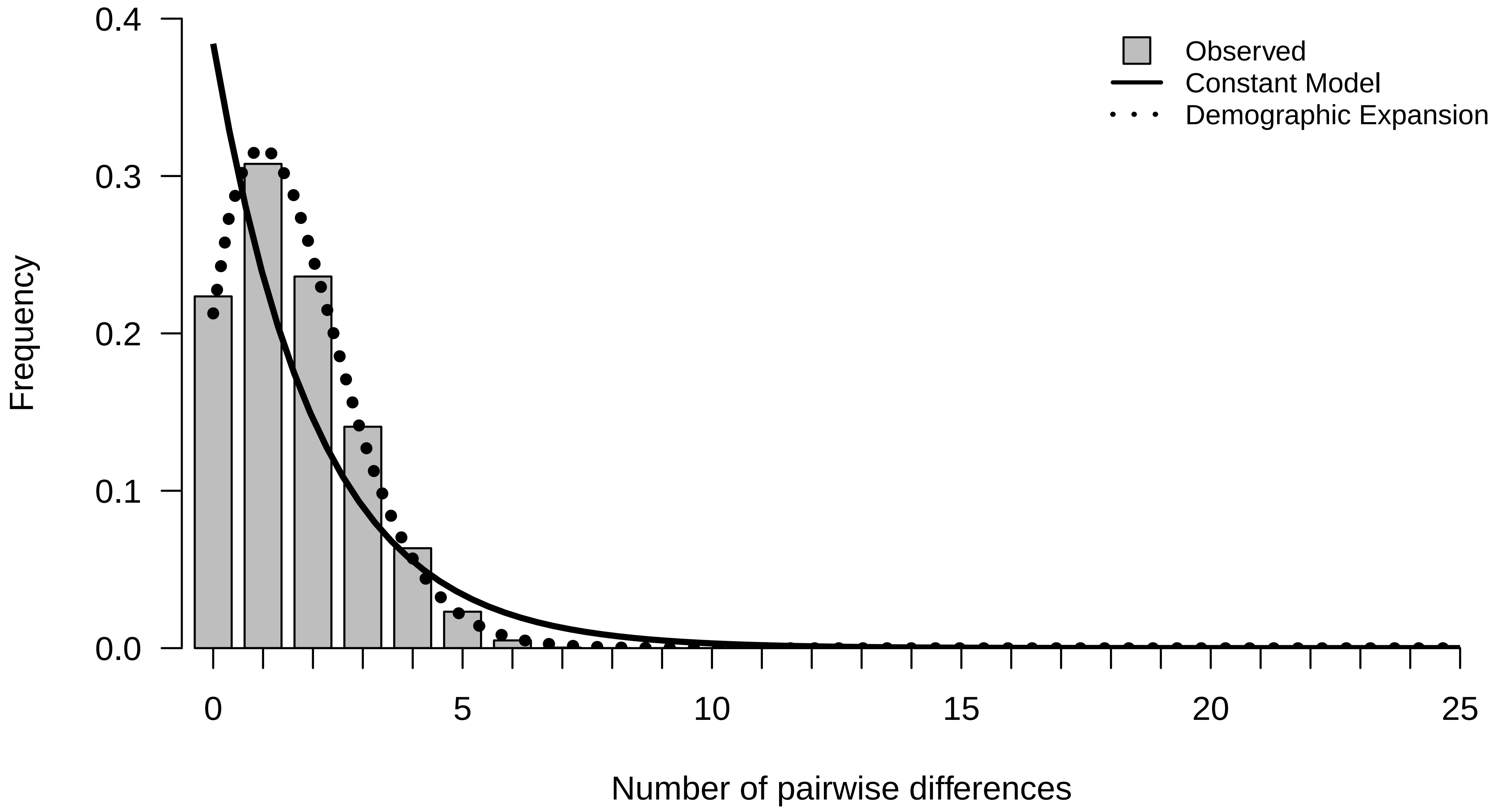

Figure 25: *Pectinaria koreni* lineage 2 mismatch analysis

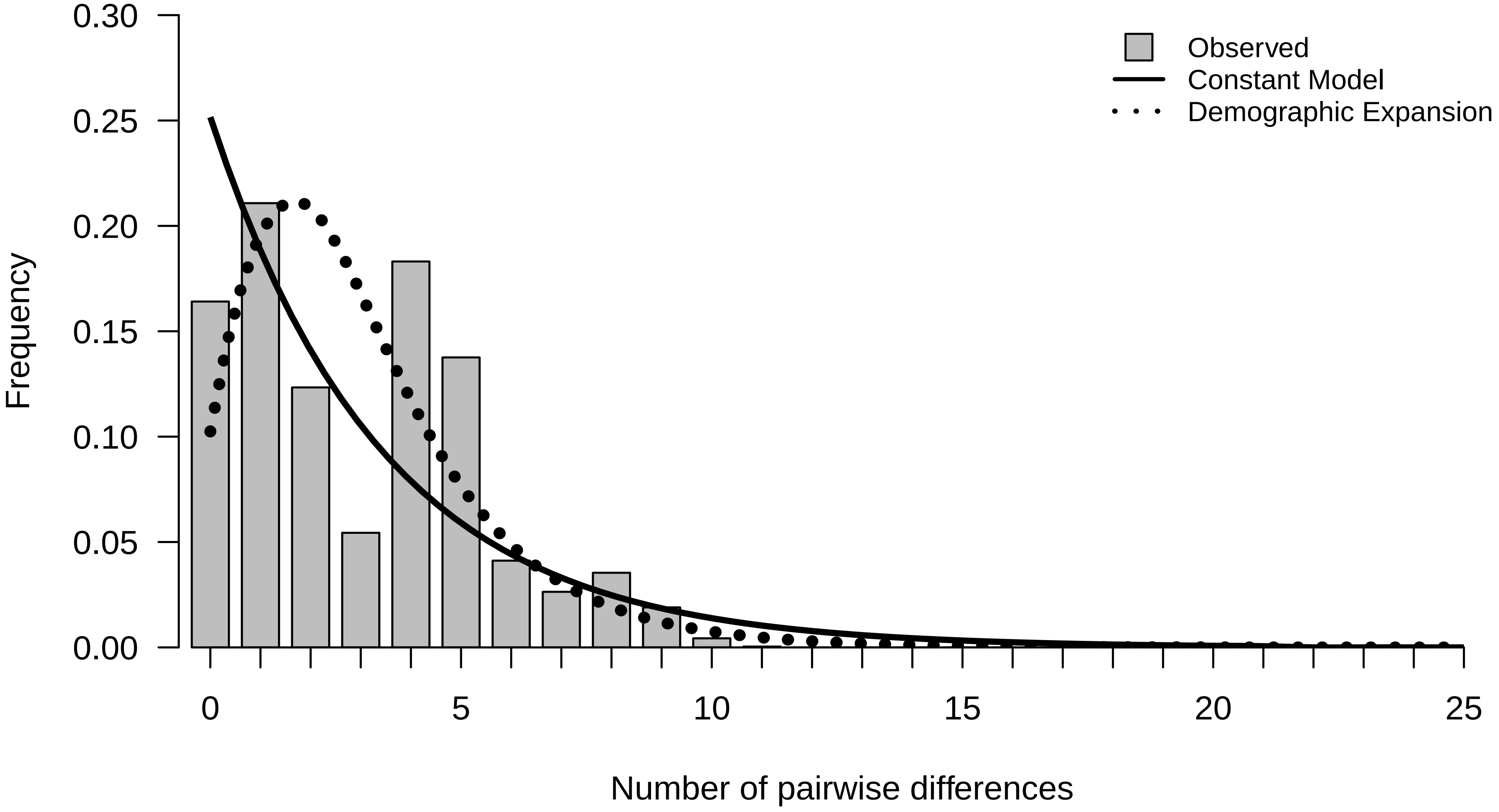

Figure 26: *Pelvetia canaliculata* mismatch analysis

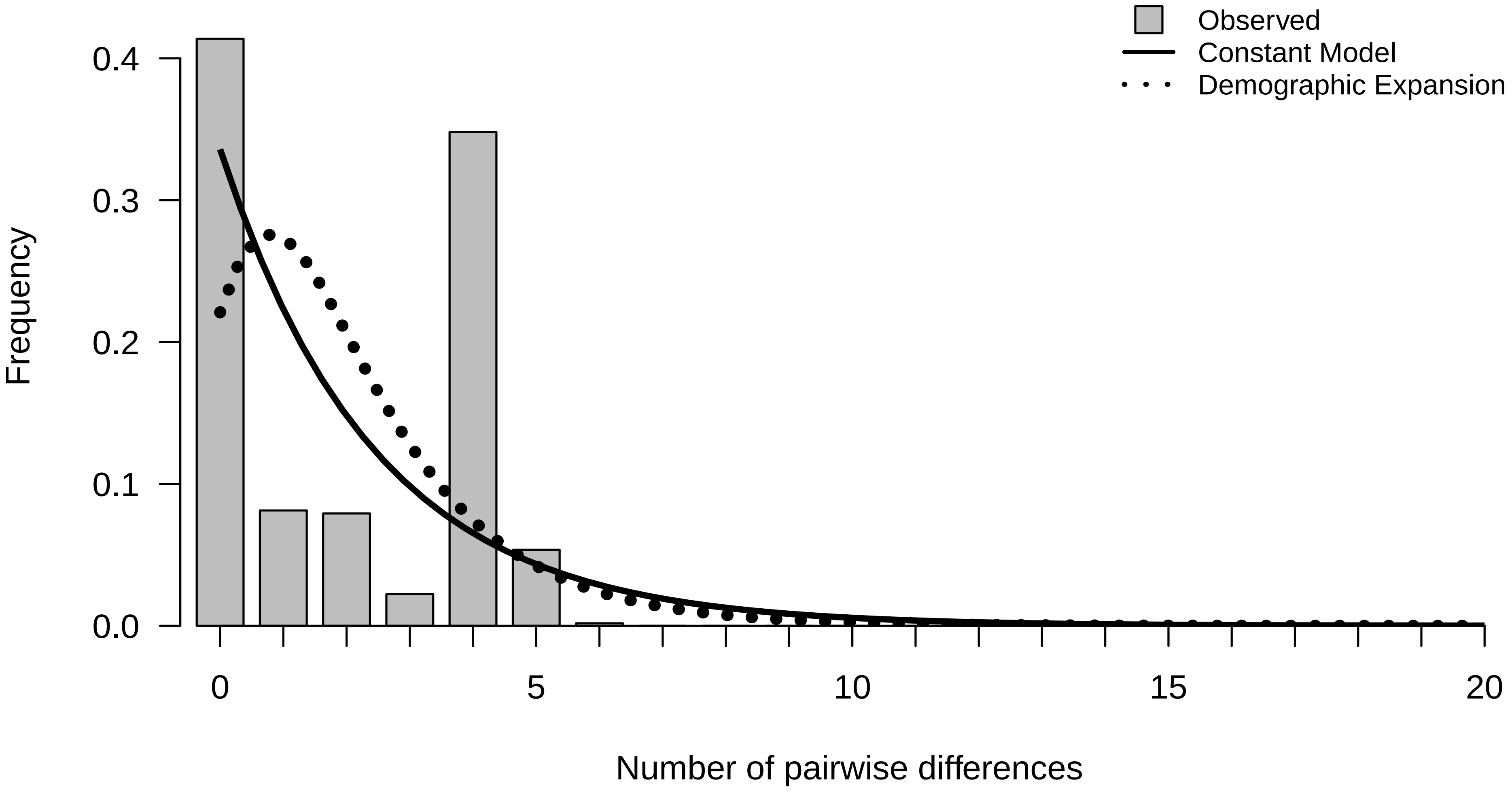

Figure 27: *Raja clavata* mismatch analysis

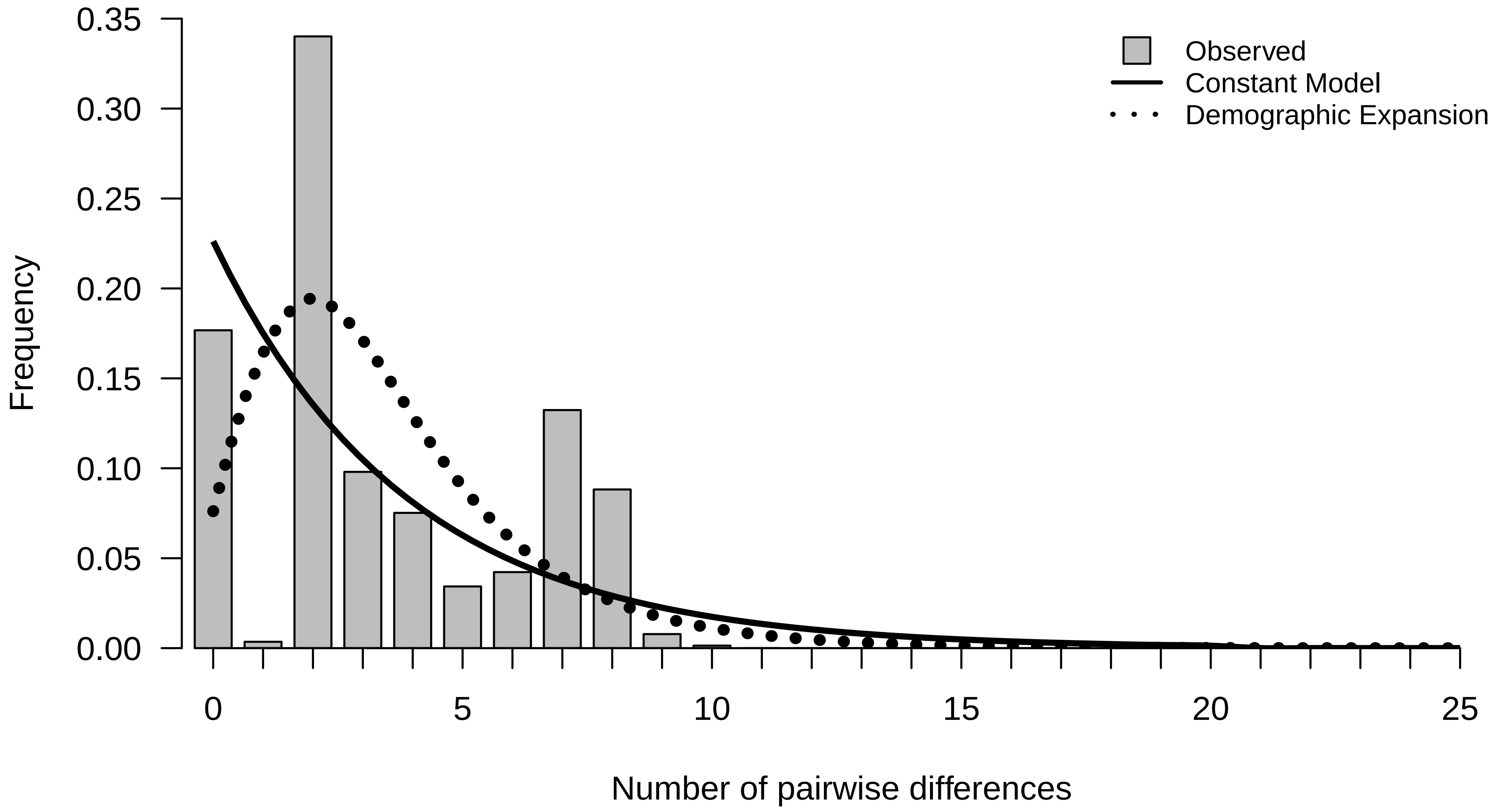

Figure 28: *Solea solea* mismatch analysis

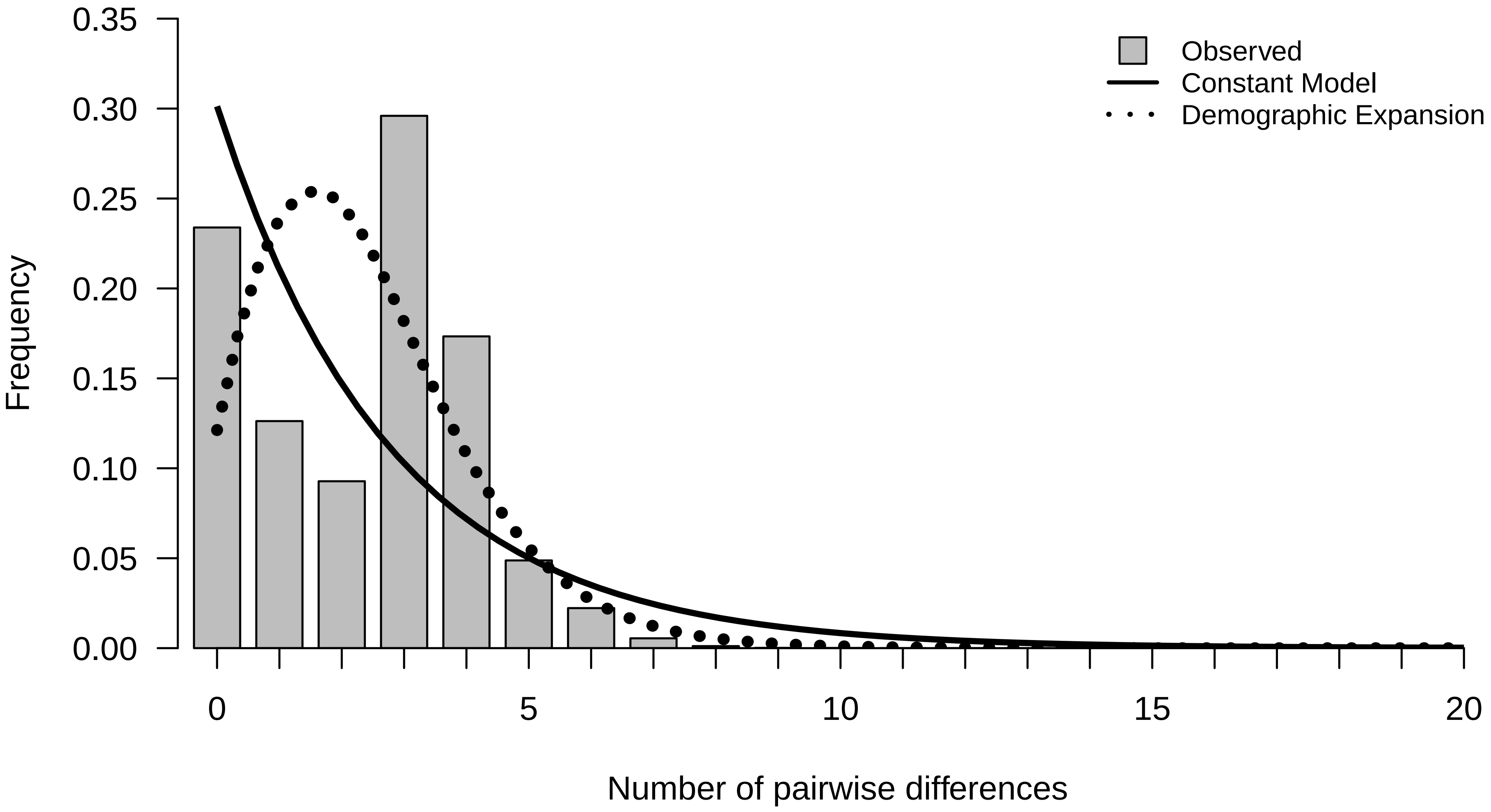

Figure 29: *Symphodus melops* mismatch analysis
